# Supplementary material for: Variants associating with uterine leiomyoma highlight genetic background shared by various cancers and hormone-related traits
Source: Nat Commun. 2018 Sep 7;9:3636. doi: 10.1038/s41467-018-05428-6 (PMC6128903; doi:10.1038/s41467-018-05428-6)
Supplement: Supplementary file 1 — Supplementary Information [file 41467_2018_5428_MOESM1_ESM.pdf]

Supplementary Information

Variants associating with uterine leiomyoma highlight genetic background

shared by various cancers and hormone-related traits

Rafnar **et al.**

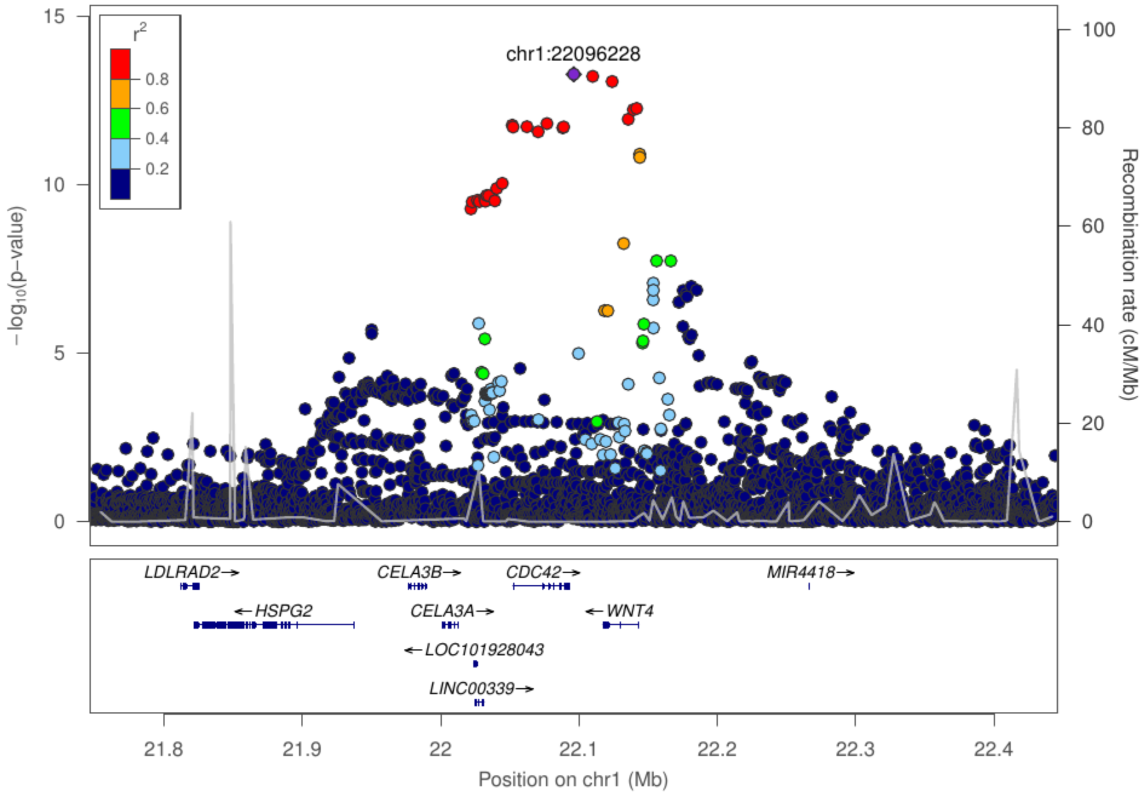
chr1:22096228
r2
0.8
0.6
0.4
0.2
−log10(p−value)
Recombination rate (cM/Mb)
LDLRAD2→
HSPG2
CELA3B→
CELA3A→
LOC101928043
LINC00339→
CDC42→
WNT4
MIR4418→
21.8
21.9
22
22.1
22.2
22.3
22.4
Position on chr1 (Mb)

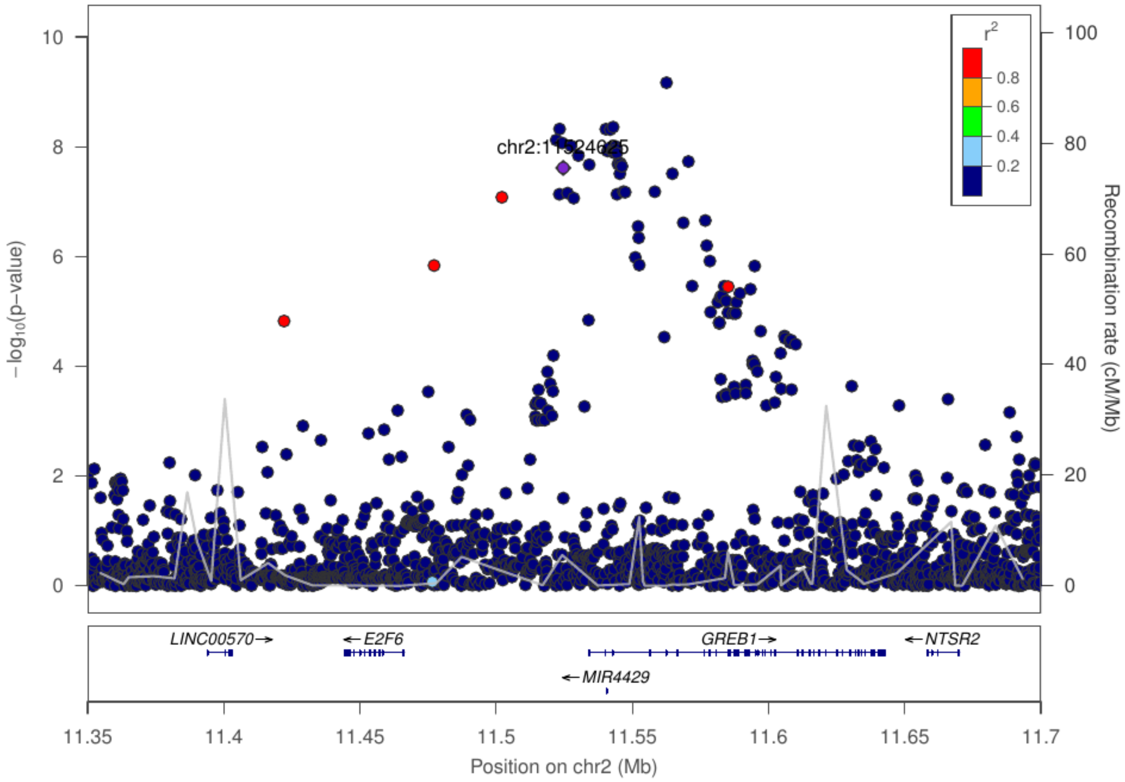
chr2:11524625
r2
0.8
0.6
0.4
0.2
−log10(p−value)
Recombination rate (cM/Mb)
LINC00570→
E2F6
GREB1→
MIR4429
NTSR2
11.35
11.4
11.45
11.5
11.55
11.6
11.65
11.7
Position on chr2 (Mb)

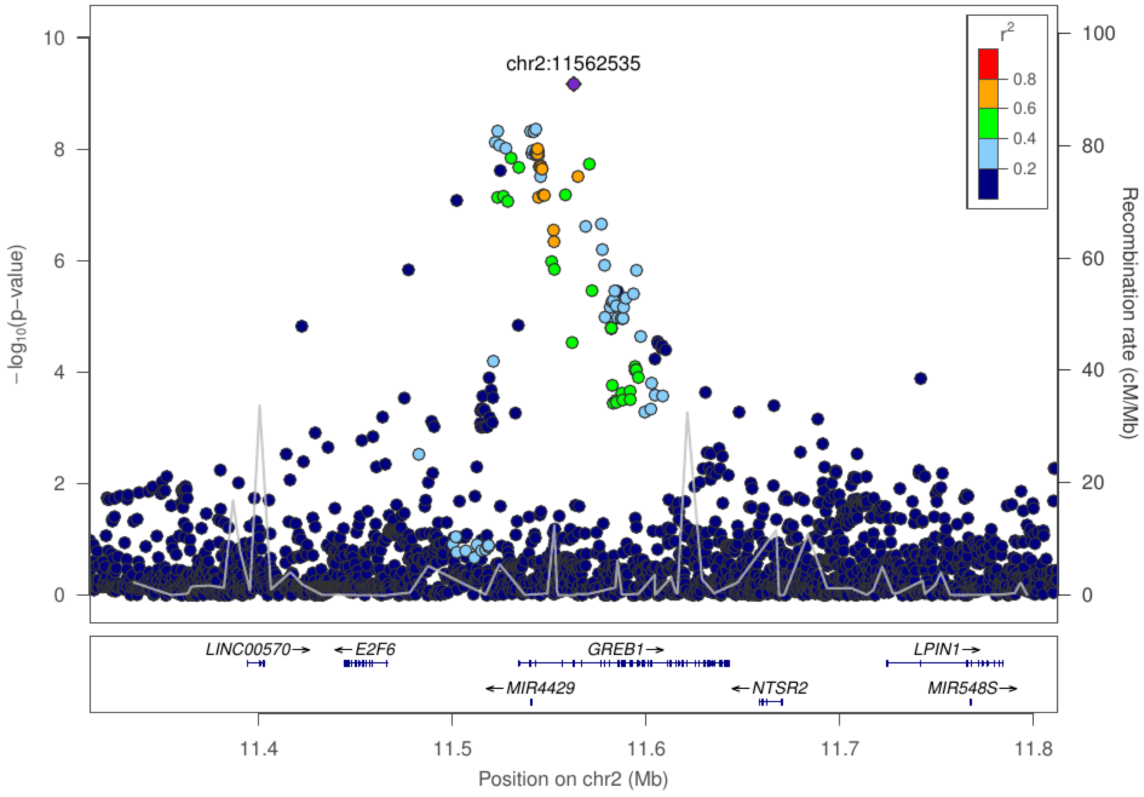
chr2:11562535
r2
0.8
0.6
0.4
0.2
−log10(p−value)
Recombination rate (cM/Mb)
LINC00570→
←E2F6
GREB1→
LPIN1→
←MIR4429
←NTSR2
MIR548S→
Position on chr2 (Mb)

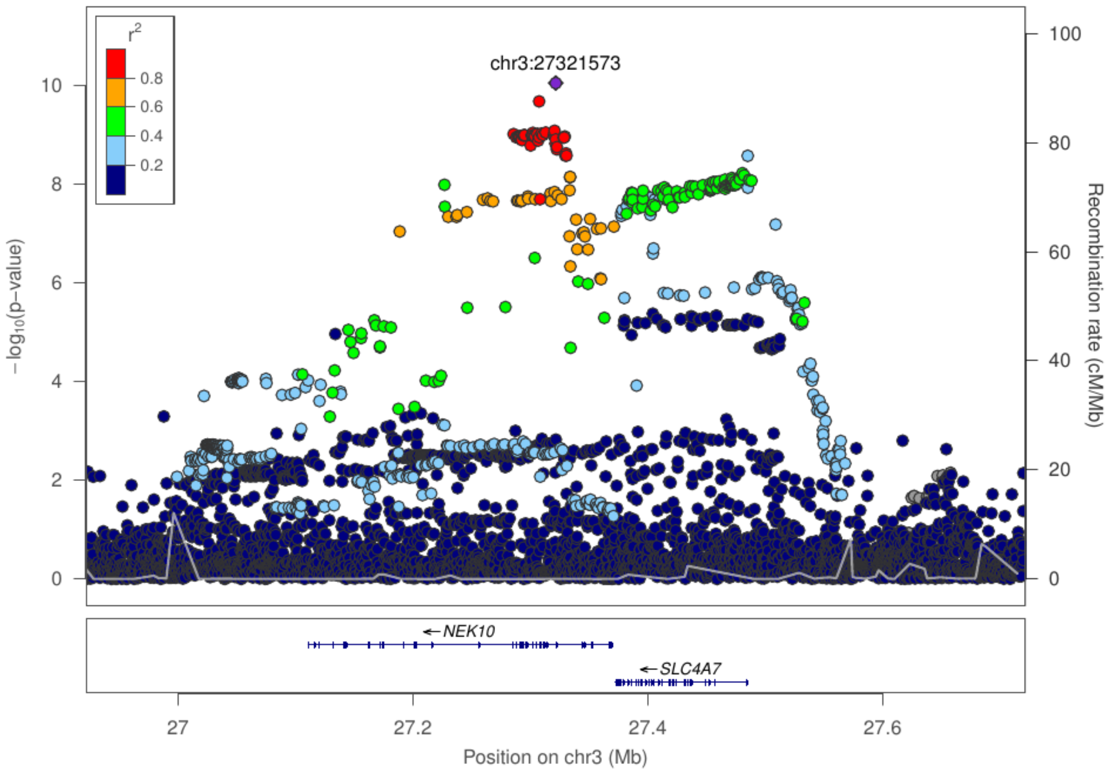
chr3:27321573
r2
0.8
0.6
0.4
0.2
−log10(p−value)
Recombination rate (cM/Mb)
←NEK10
←SLC4A7
Position on chr3 (Mb)

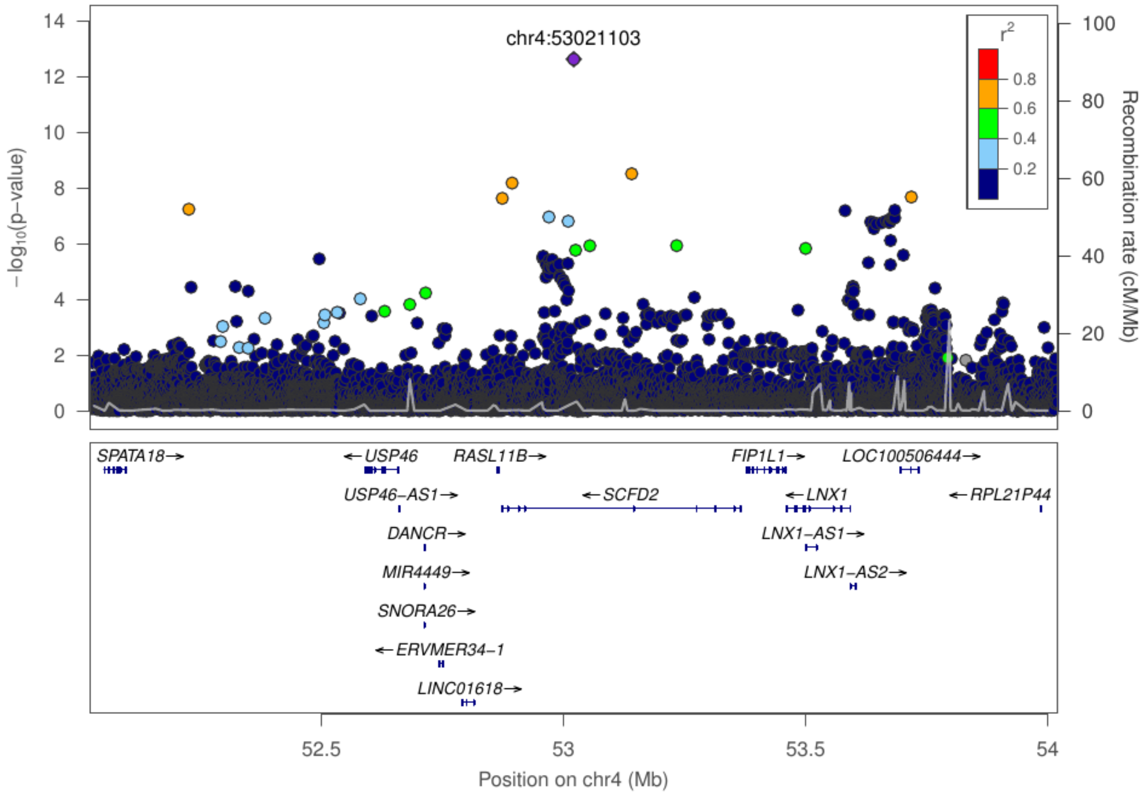
chr4:53021103
r2
0.8
0.6
0.4
0.2
− log10(p−value)
Recombination rate (cM/Mb)
SPATA18
USP46
RASL11B
FIP1L1
LOC100506444
USP46−AS1
SCFD2
LNX1
RPL21P44
DANCR
LNX1−AS1
MIR4449
LNX1−AS2
SNORA26
ERVMER34−1
LINC01618
Position on chr4 (Mb)

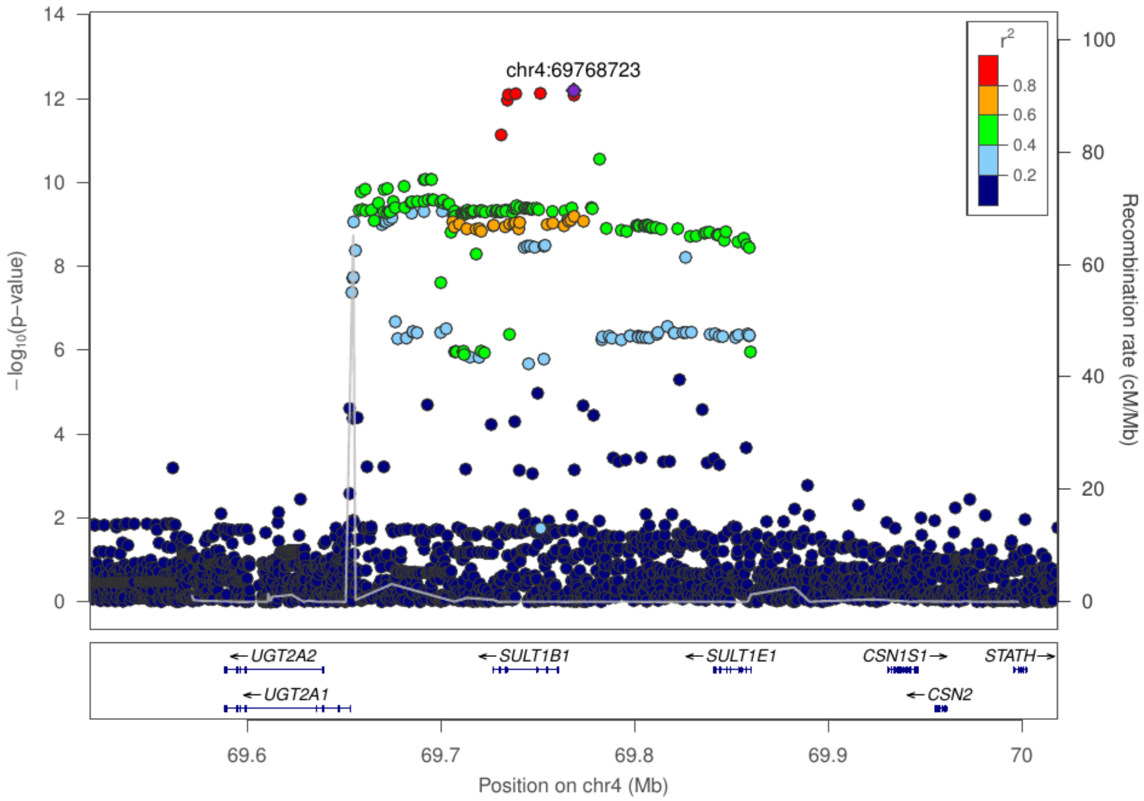
chr4:69768723
r2
0.8
0.6
0.4
0.2
− log10(p−value)
Recombination rate (cM/Mb)
UGT2A2
SULT1B1
SULT1E1
CSN1S1
STATH
UGT2A1
CSN2
Position on chr4 (Mb)

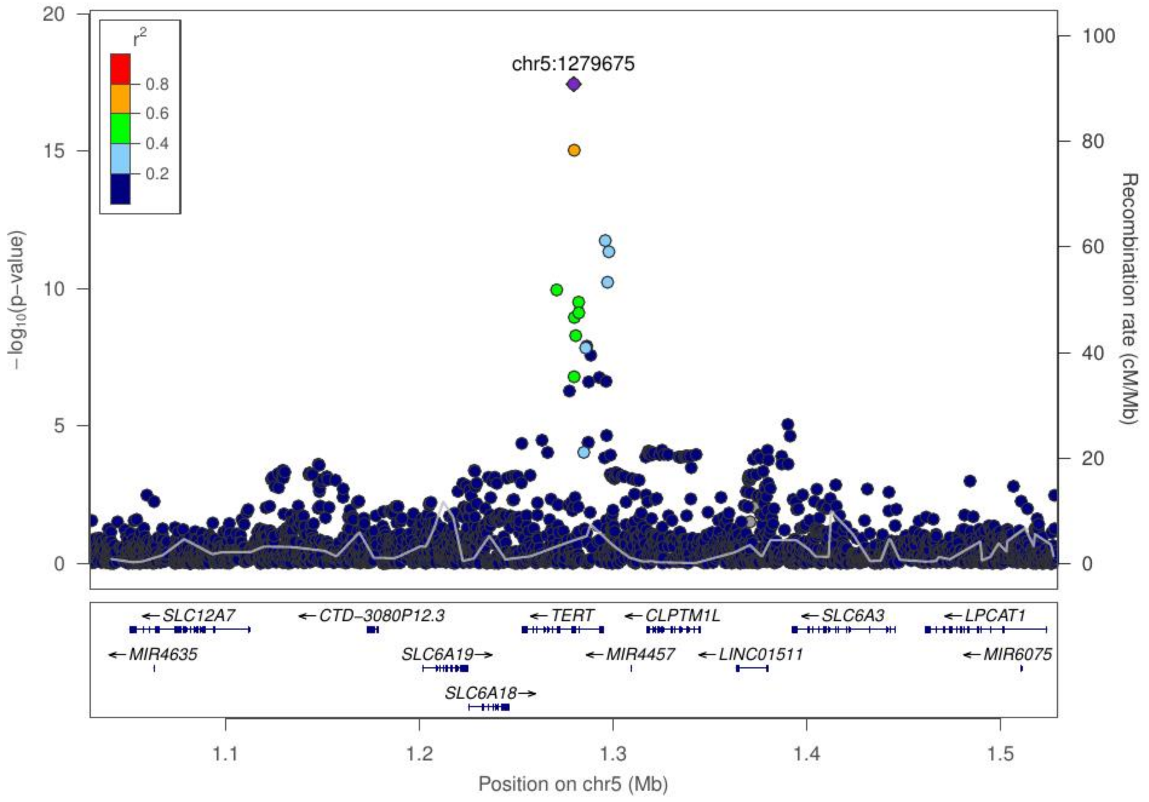
chr5:1279675
r2
0.8
0.6
0.4
0.2
− log10(p−value)
Recombination rate (cM/Mb)
SLC12A7
CTD−3080P12.3
TERT
CLPTM1L
SLC6A3
LPCAT1
MIR4635
SLC6A19
MIR4457
LINC01511
MIR6075
SLC6A18
Position on chr5 (Mb)

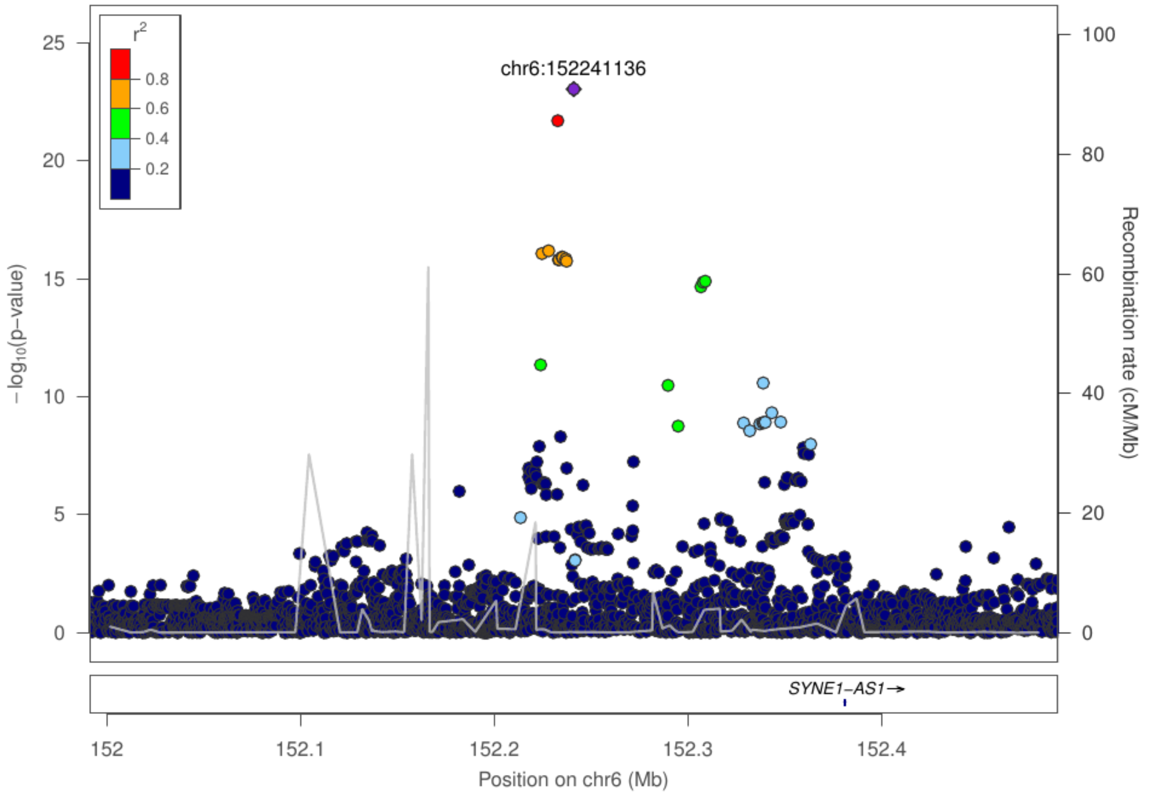
chr6:152241136
r2
0.8
0.6
0.4
0.2
− log10(p−value)
Recombination rate (cM/Mb)
SYNE1−AS1
Position on chr6 (Mb)

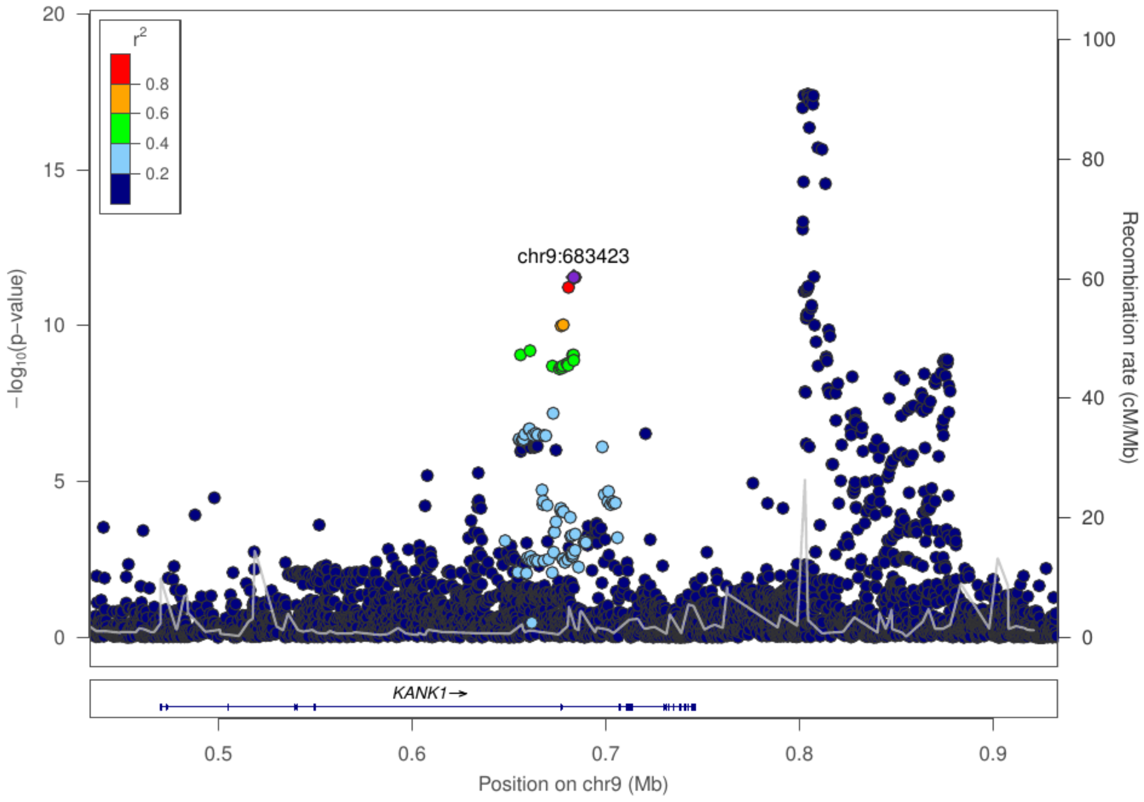

chr9:683423
r2
0.8
0.6
0.4
0.2
− log10(p−value)
Recombination rate (cM/Mb)
KANK1→
Position on chr9 (Mb)

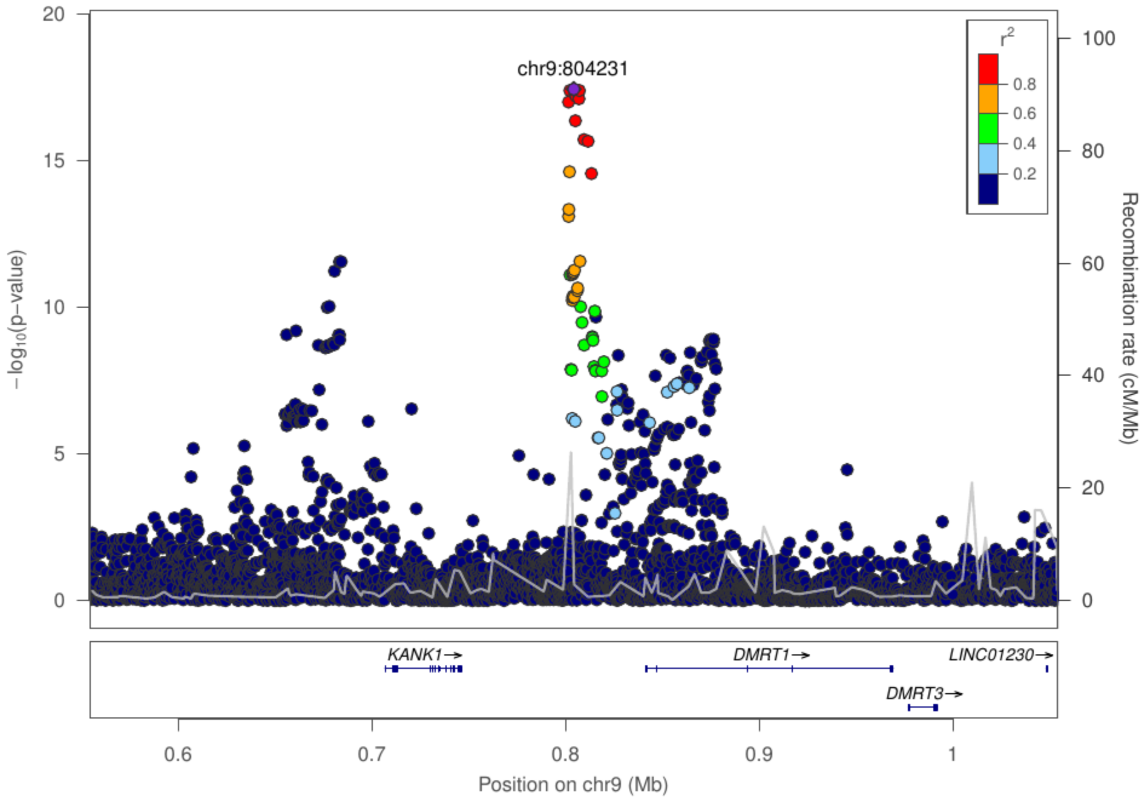

chr9:804231
r2
0.8
0.6
0.4
0.2
− log10(p−value)
Recombination rate (cM/Mb)
KANK1→
DMRT1→
LINC01230→
DMRT3→
Position on chr9 (Mb)

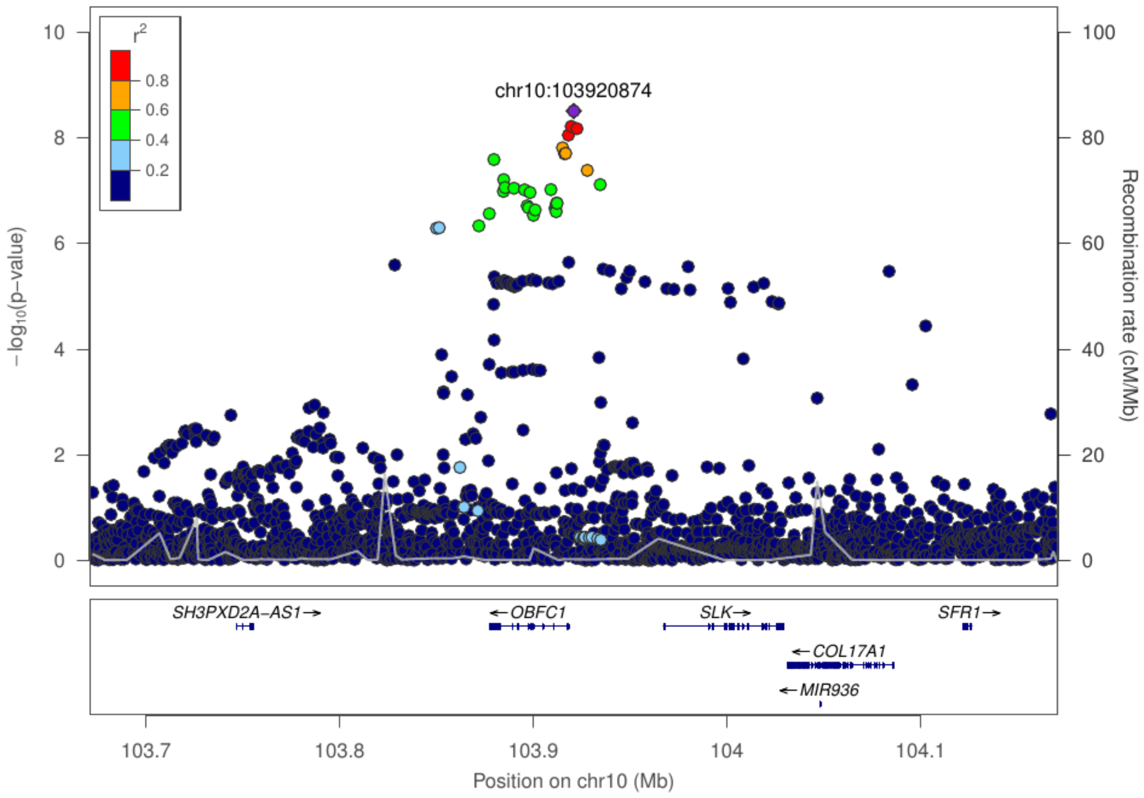
chr10:103920874
r2
0.8
0.6
0.4
0.2
−log10(p−value)
Recombination rate (cM/Mb)
SH3PXD2A−AS1→
←OBFC1
SLK→
SFR1→
←COL17A1
←MIR936
103.7
103.8
103.9
104
104.1
Position on chr10 (Mb)

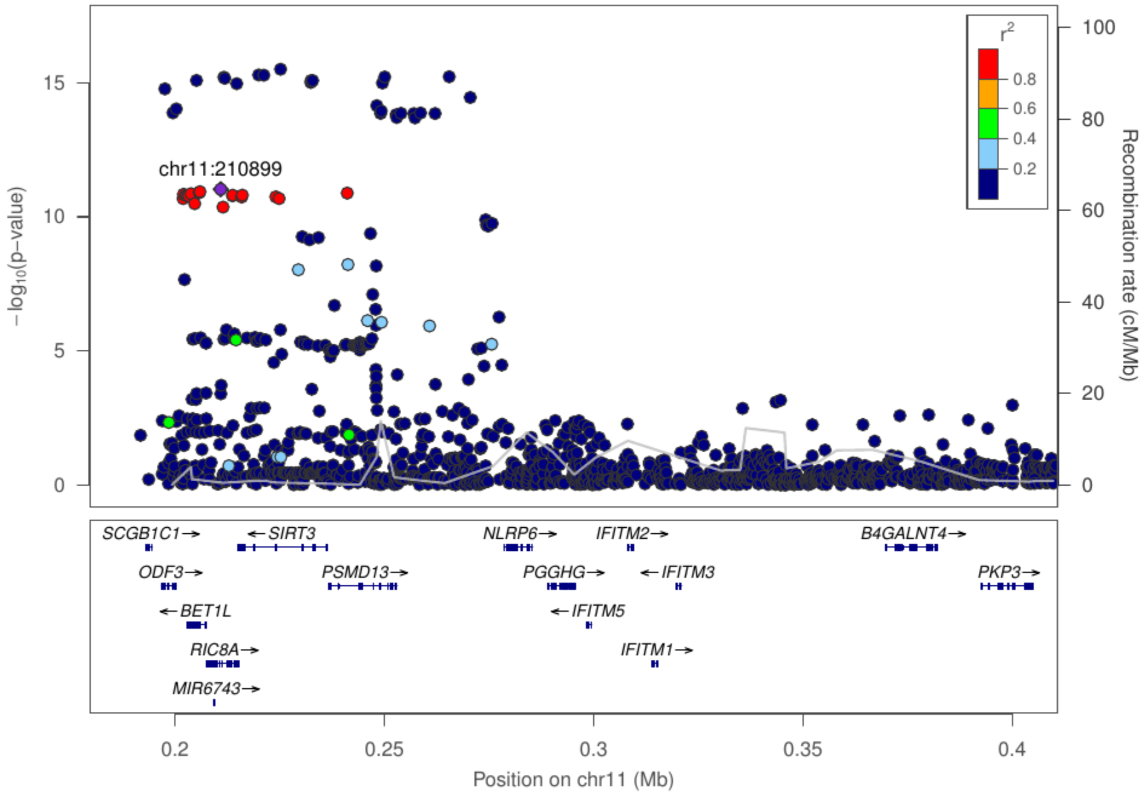
chr11:210899
r2
0.8
0.6
0.4
0.2
−log10(p−value)
Recombination rate (cM/Mb)
SCGB1C1→
←SIRT3
NLRP6→
IFITM2→
B4GALNT4→
ODF3→
PSMD13→
PGGHG→
←IFITM3
PKP3→
←BET1L
←IFITM5
RIC8A→
IFITM1→
MIR6743→
0.2
0.25
0.3
0.35
0.4
Position on chr11 (Mb)

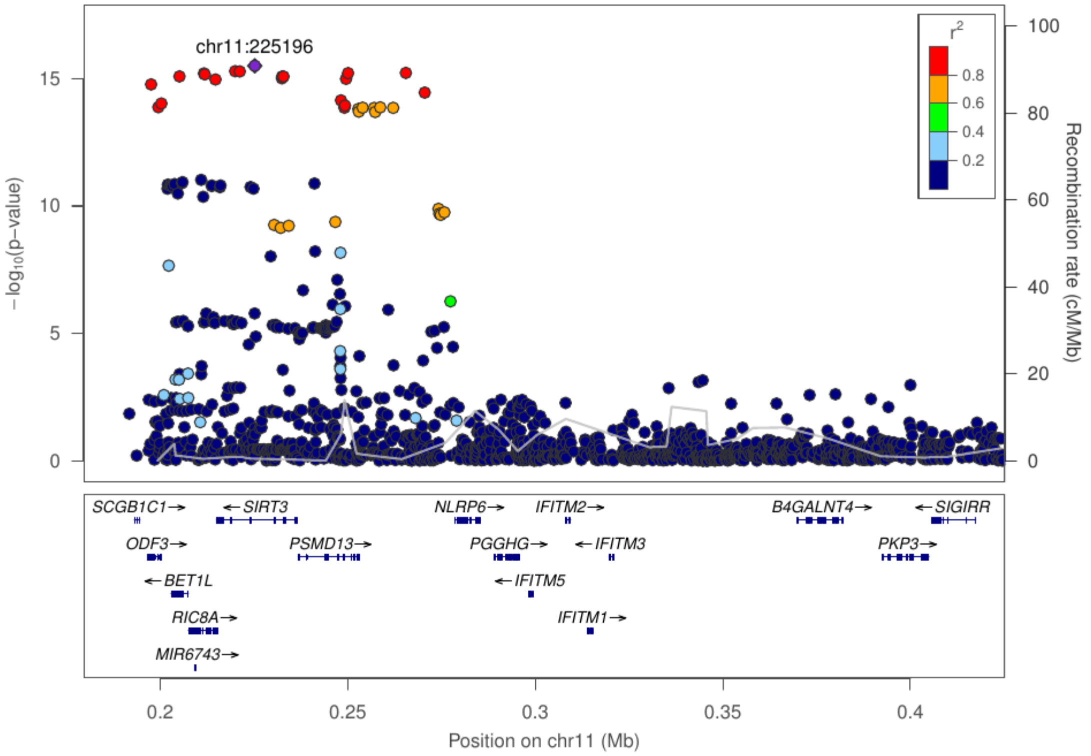
chr11:225196
r2
0.8
0.6
0.4
0.2
−log10(p−value)
Recombination rate (cM/Mb)
SCGB1C1
SIRT3
NLRP6
IFITM2
B4GALNT4
SIGIRR
ODF3
PSMD13
PGGHG
IFITM3
PKP3
BET1L
IFITM5
RIC8A
IFITM1
MIR6743
Position on chr11 (Mb)

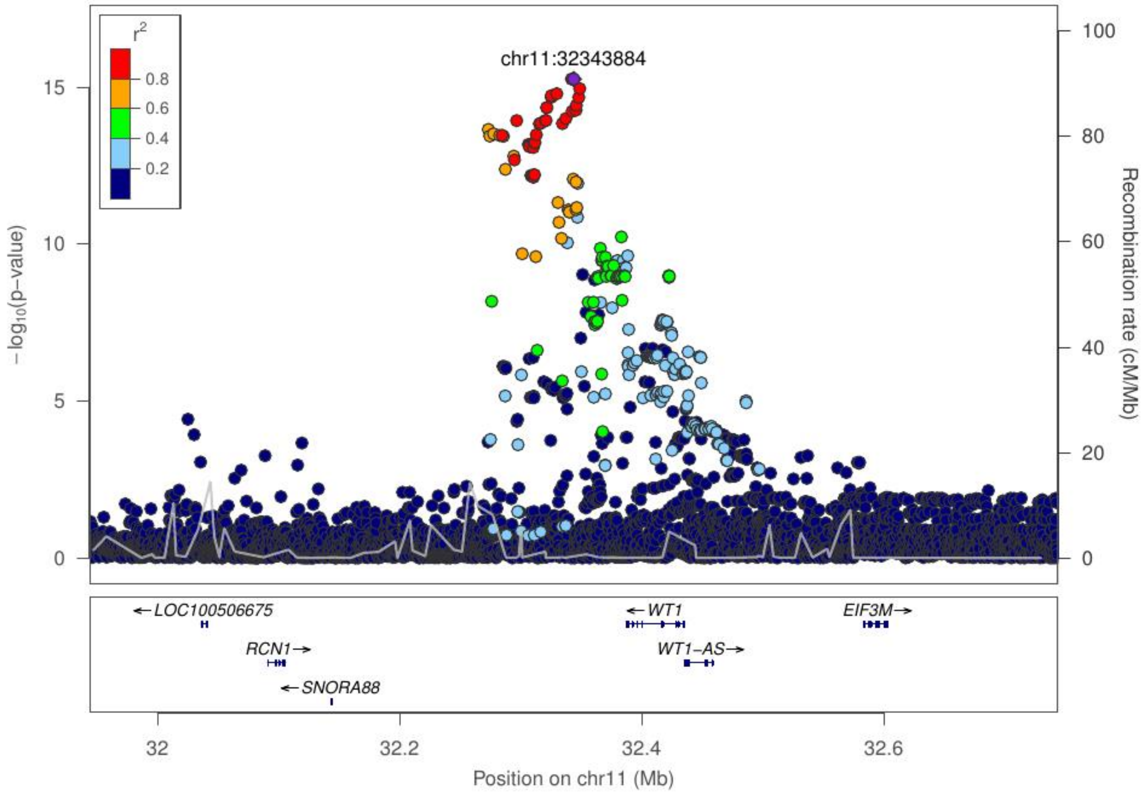
chr11:32343884
r2
0.8
0.6
0.4
0.2
−log10(p−value)
Recombination rate (cM/Mb)
LOC100506675
WT1
EIF3M
RCN1
WT1−AS
SNORA88
Position on chr11 (Mb)

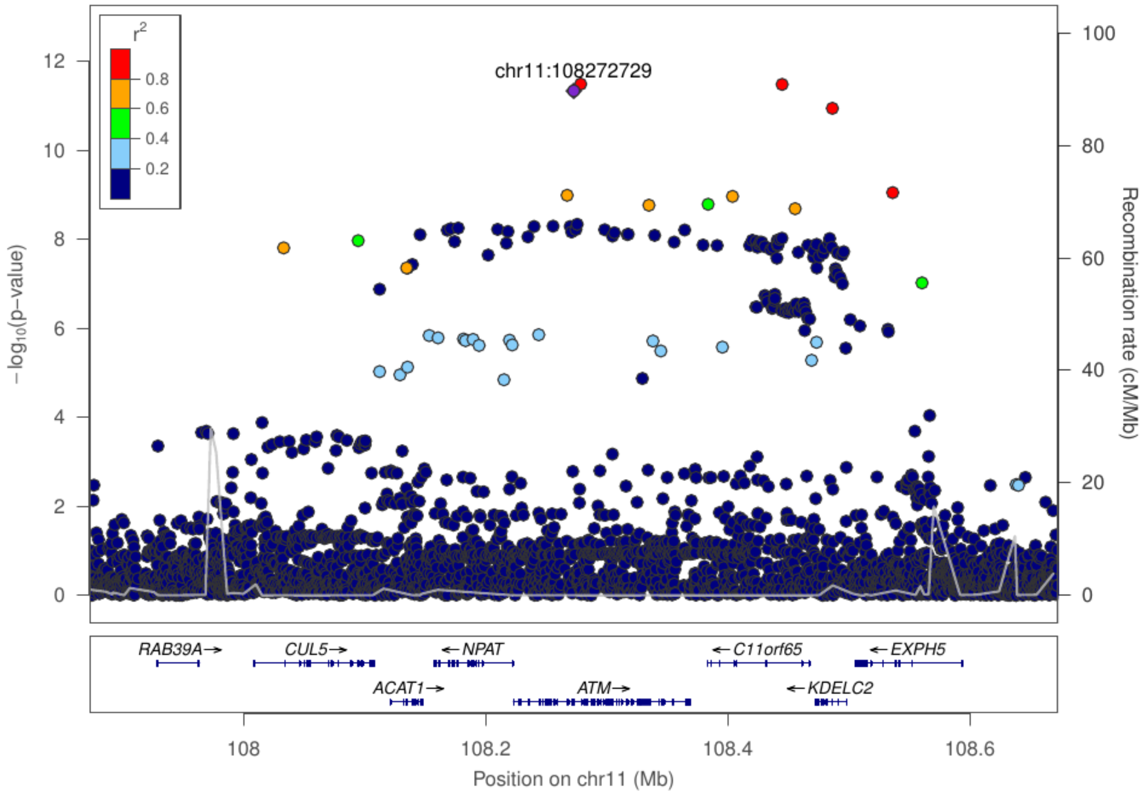

r2
0.8
0.6
0.4
0.2
chr11:108272729
−log10(p−value)
Recombination rate (cM/Mb)
RAB39A
CUL5
NPAT
C11orf65
EXPH5
ACAT1
ATM
KDELC2
108
108.2
108.4
108.6
Position on chr11 (Mb)

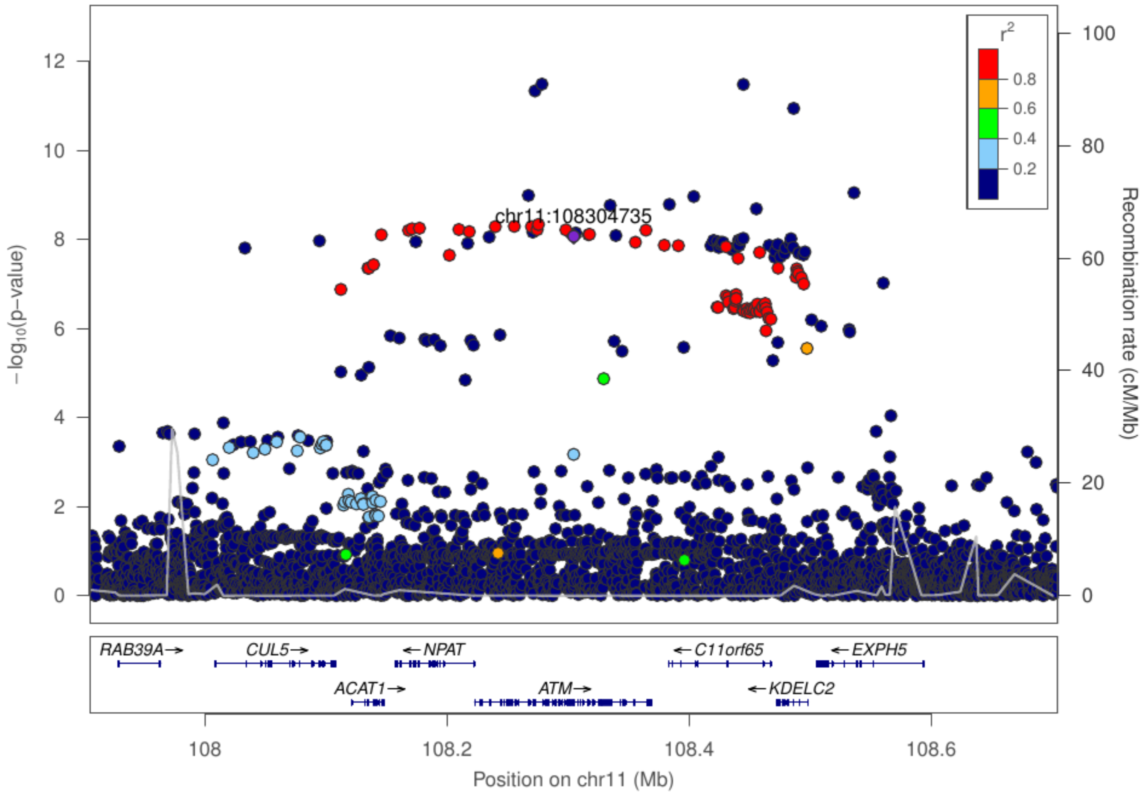

r2
0.8
0.6
0.4
0.2
chr11:108304735
−log10(p−value)
Recombination rate (cM/Mb)
RAB39A
CUL5
NPAT
C11orf65
EXPH5
ACAT1
ATM
KDELC2
108
108.2
108.4
108.6
Position on chr11 (Mb)

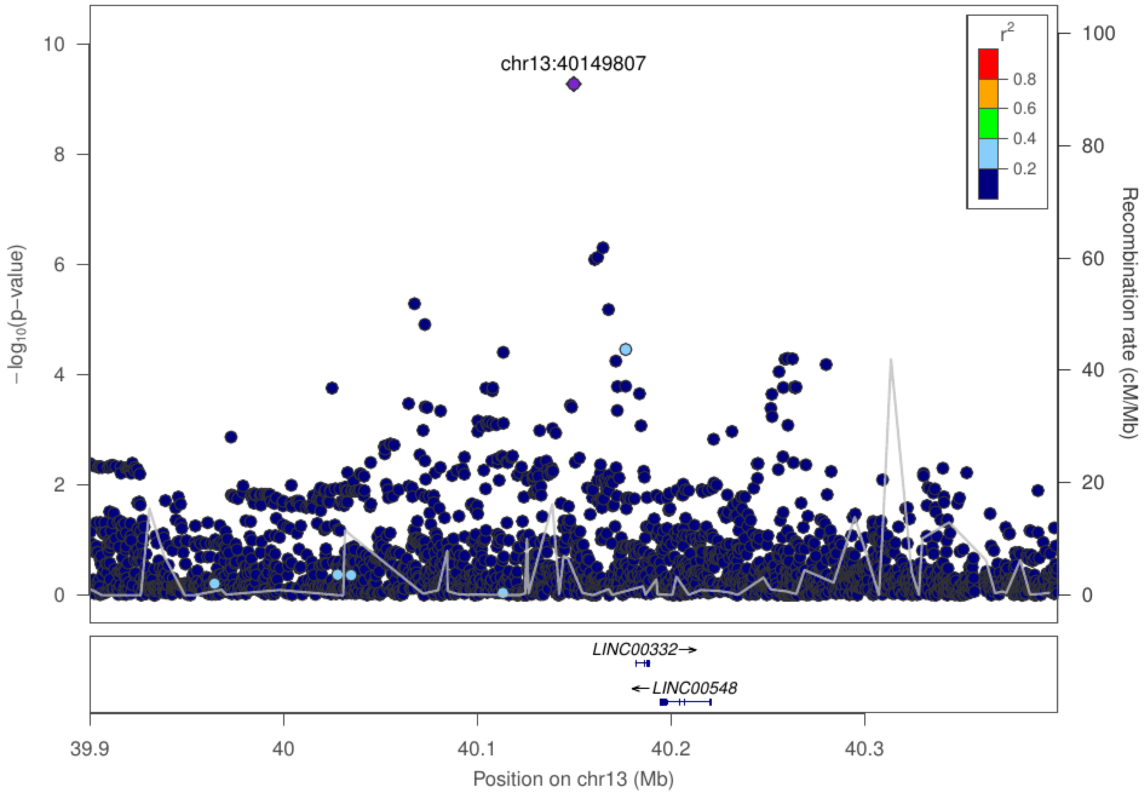
chr13:40149807
r2
0.8
0.6
0.4
0.2
−log10(p−value)
Recombination rate (cM/Mb)
LINC00332
LINC00548
39.9
40
40.1
40.2
40.3
Position on chr13 (Mb)

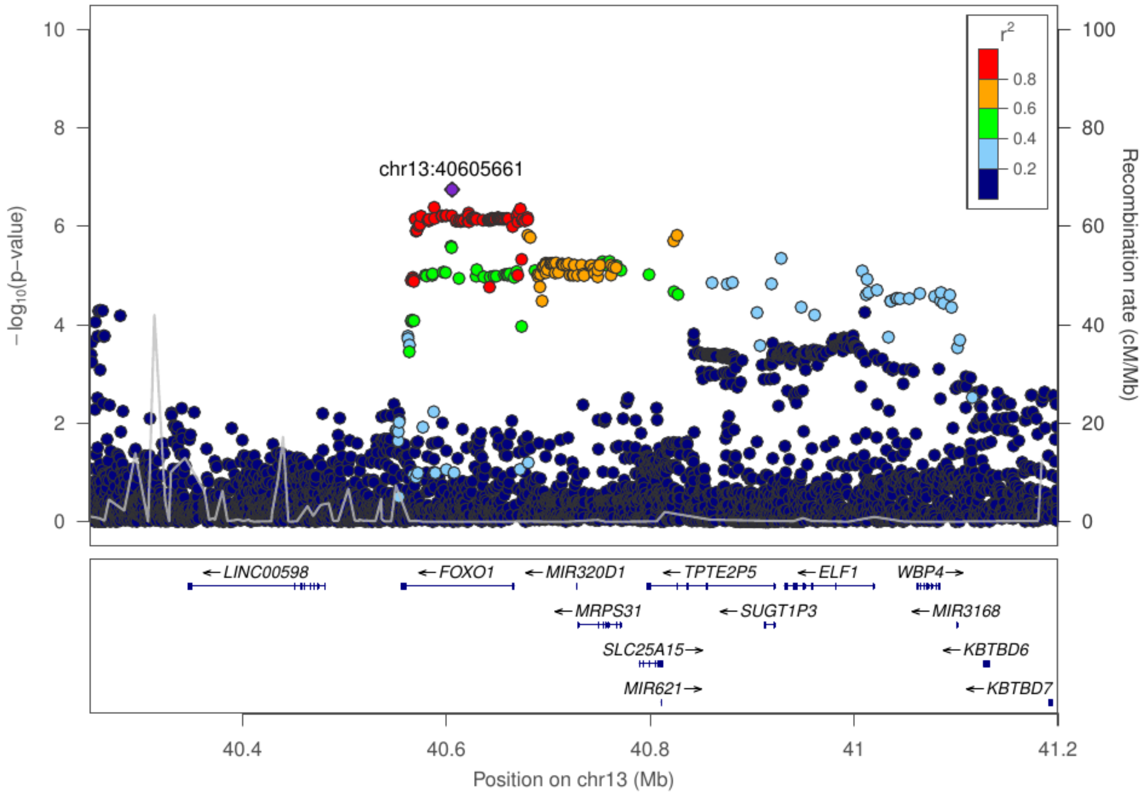
chr13:40605661
r2
0.8
0.6
0.4
0.2
−log10(p−value)
Recombination rate (cM/Mb)
LINC00598
FOXO1
MIR320D1
TPTE2P5
ELF1
WBP4
MRPS31
SUGT1P3
MIR3168
SLC25A15
KBTBD6
MIR621
KBTBD7
40.4
40.6
40.8
41
41.2
Position on chr13 (Mb)

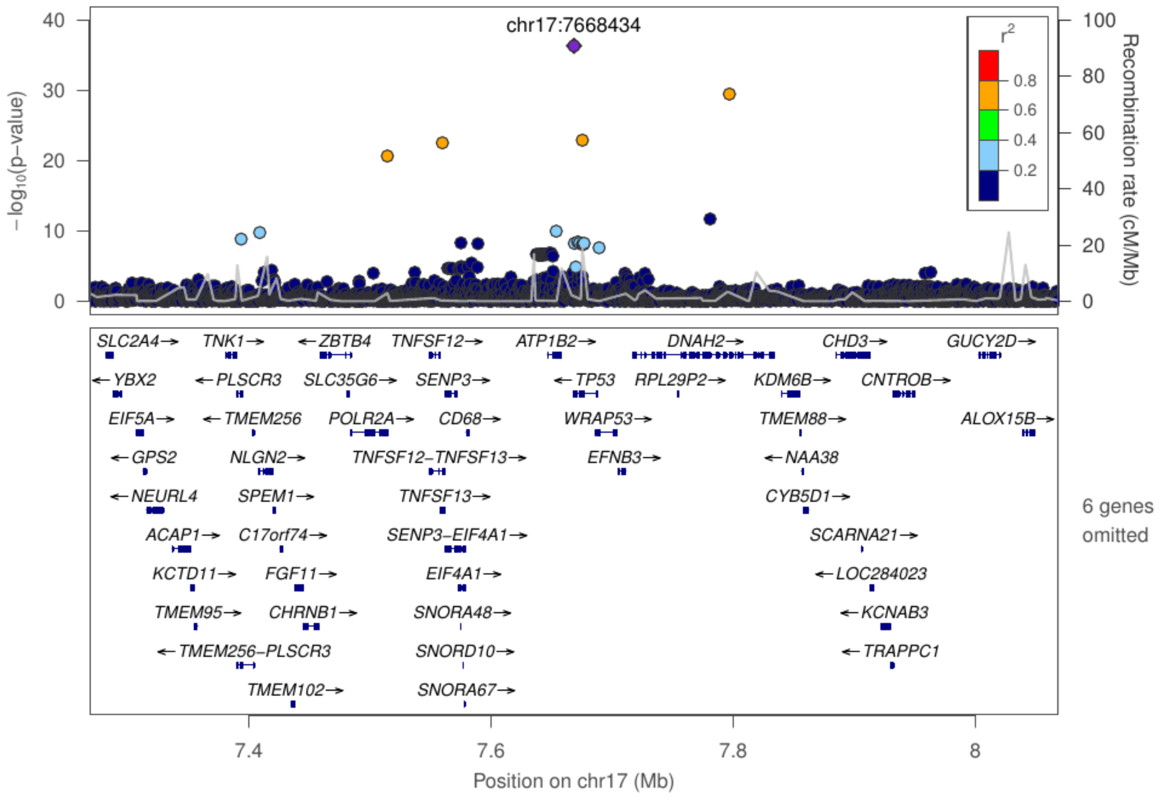
chr17:7668434
r2
0.8
0.6
0.4
0.2
− log10(p−value)
Recombination rate (cM/Mb)
SLC2A4→ TNK1→ ←ZBTB4 TNFSF12→ ATP1B2→ DNAH2→ CHD3→ GUCY2D→
←YBX2 ←PLSCR3 SLC35G6→ SENP3→ ←TP53 RPL29P2→ KDM6B→ CNTROB→
EIF5A→ ←TMEM256 POLR2A→ CD68→ WRAP53→ TMEM88→ ALOX15B→
←GPS2 NLGN2→ TNFSF12−TNFSF13→ EFNB3→ ←NAA38
←NEURL4 SPEM1→ TNFSF13→ CYB5D1→
ACAP1→ C17orf74→ SENP3−EIF4A1→ SCARNA21→
KCTD11→ FGF11→ EIF4A1→ ←LOC284023
TMEM95→ CHRNB1→ SNORA48→ ←KCNAB3
←TMEM256−PLSCR3 SNORD10→ ←TRAPPC1
TMEM102→ SNORA67→
6 genes omitted
7.4
7.6
7.8
8
Position on chr17 (Mb)

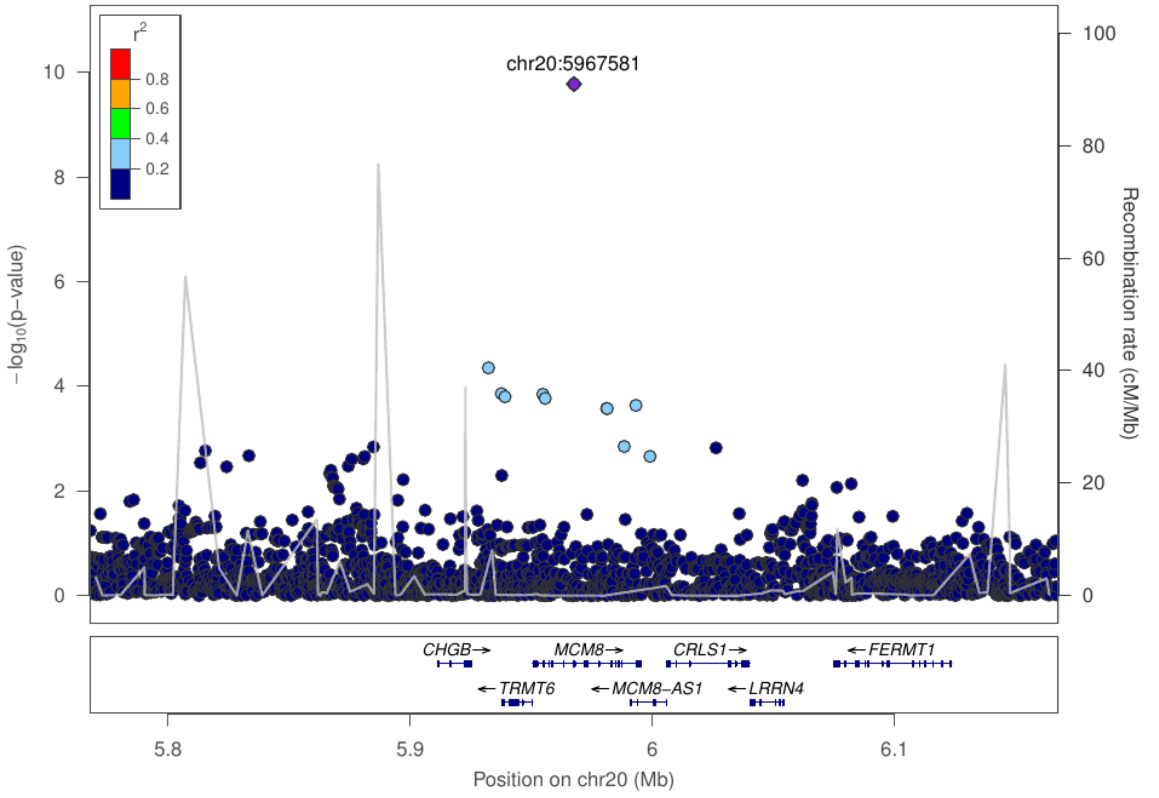
chr20:5967581
r2
0.8
0.6
0.4
0.2
− log10(p−value)
Recombination rate (cM/Mb)
CHGB→ MCM8→ CRLS1→ ←FERMT1
←TRMT6 ←MCM8−AS1 ←LRRN4
5.8
5.9
6
6.1
Position on chr20 (Mb)

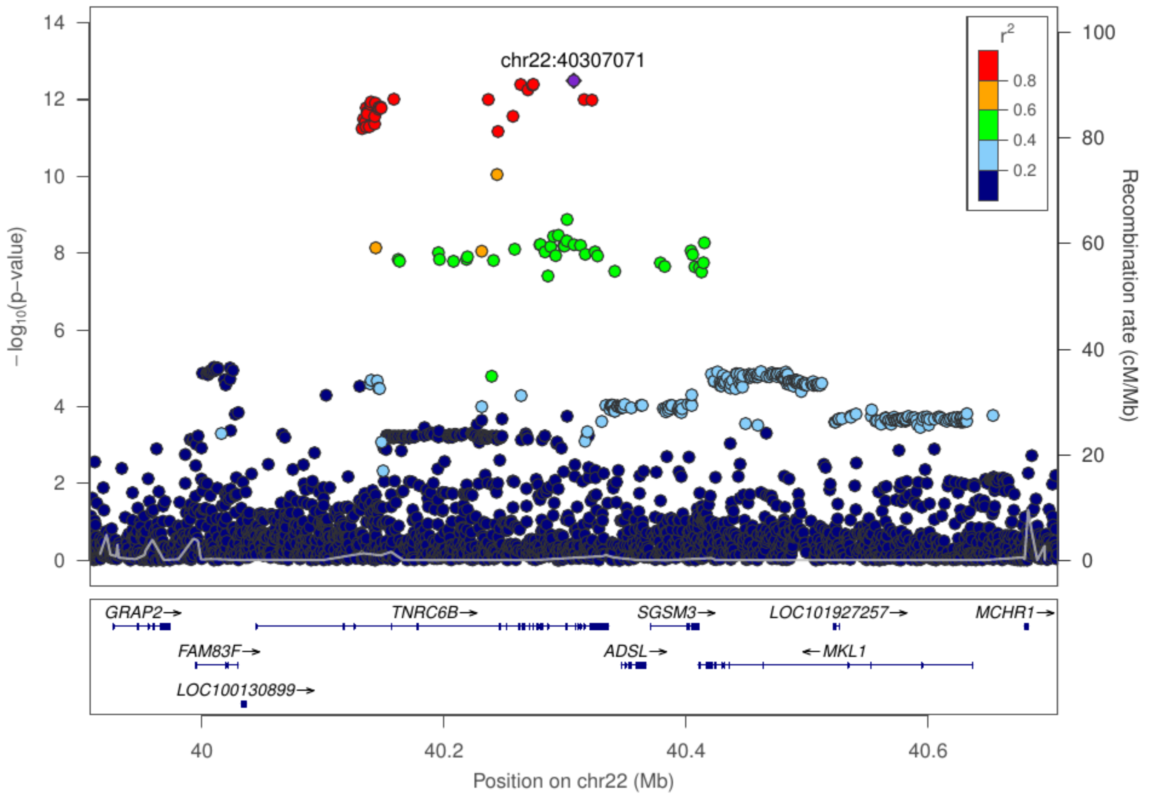

Supplementary figure 1. Locus plots for variants associating with leiomyoma from meta-analysis of Icelandic and UK Biobank GWAS datasets. P values (−log10) of variant associations with leiomyoma in the meta-analysis are plotted against their NCBI Build 38 positions. Known genes in the regions are shown underneath each plot, taken from the UCSC Genes track in the UCSC Genome Browser. Only high-quality markers are displayed with a 400kb window around the lead variant.

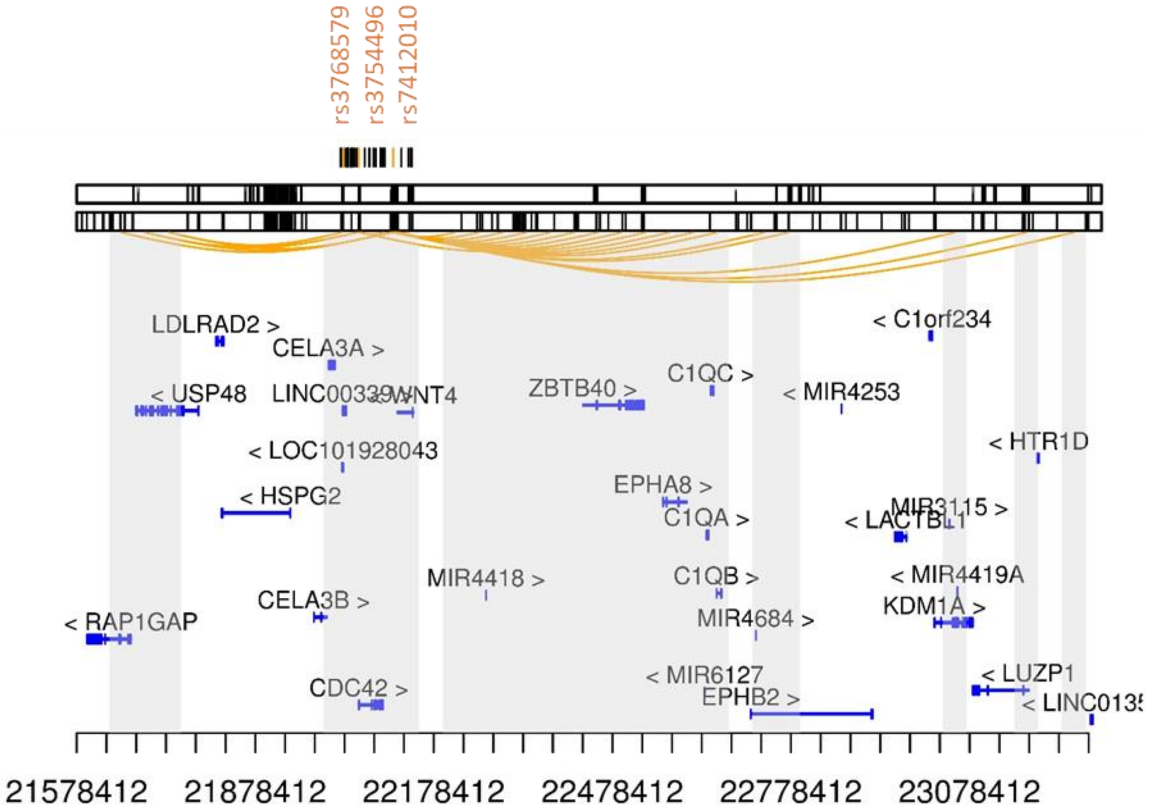
rs3768579
rs3754496
rs7412010
LDLRAD2 >
CELA3A >
< USP48
LINC00339
WNT4
< LOC101928043
< HSPG2
CELA3B >
< RAP1GAP
CDC42 >
MIR4418 >
ZBTB40 >
C1QC >
EPHA8 >
C1QA >
C1QB >
MIR4684 >
< MIR6127
EPHB2 >
< MIR4253
< C1orf234
< HTR1D
MIR3115 >
< LACTBL1
< MIR4419A
KDM1A >
< LUZP1
21578412
21878412
22178412
22478412
22778412
23078412

chr1

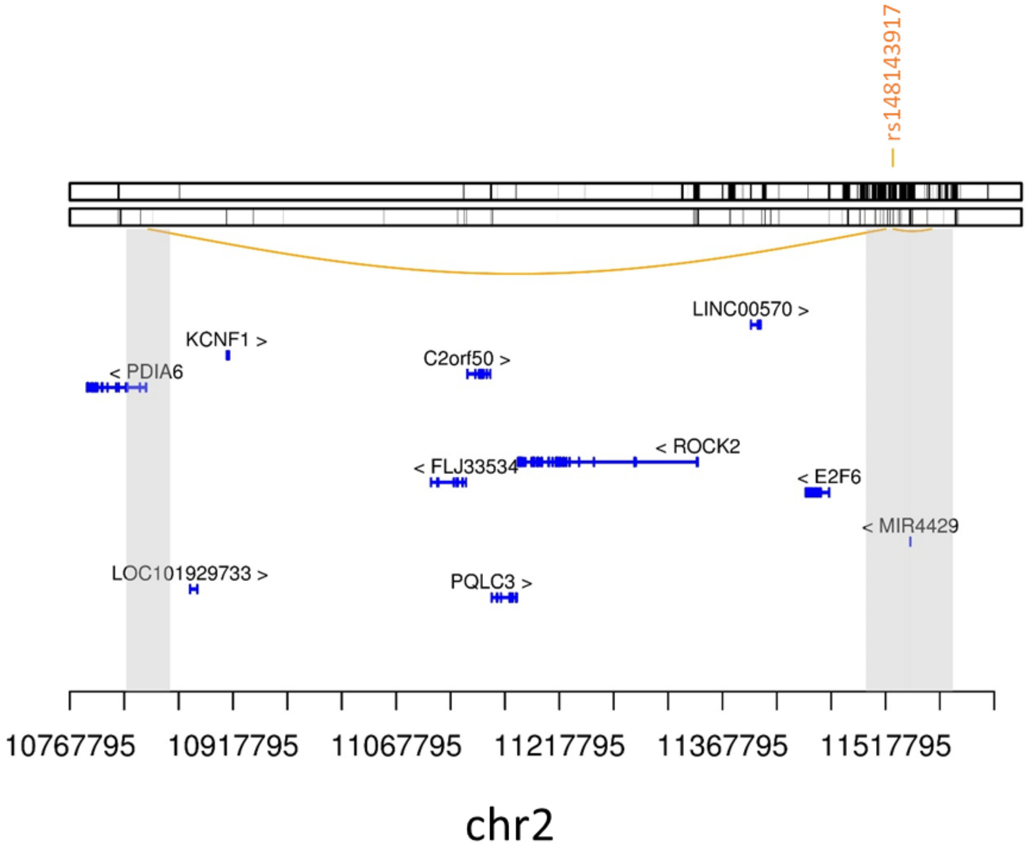

rs148143917
LINC00570 >
KCNF1 >
C2orf50 >
< PDIA6
< ROCK2
< FLJ33534
< E2F6
< MIR4429
LOC101929733 >
PQLC3 >
10767795
10917795
11067795
11217795
11367795
11517795
chr2

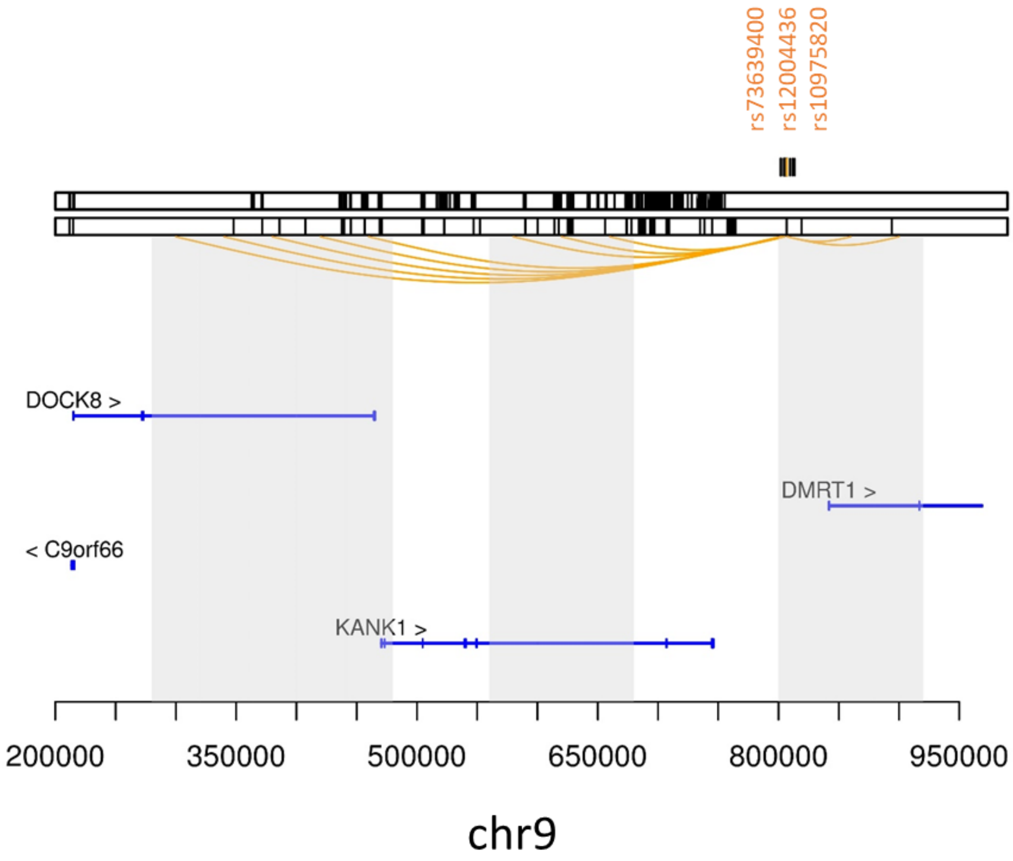
rs73639400
rs12004436
rs10975820
DOCK8 >
DMRT1 >
< C9orf66
KANK1 >
200000
350000
500000
650000
800000
950000
chr9

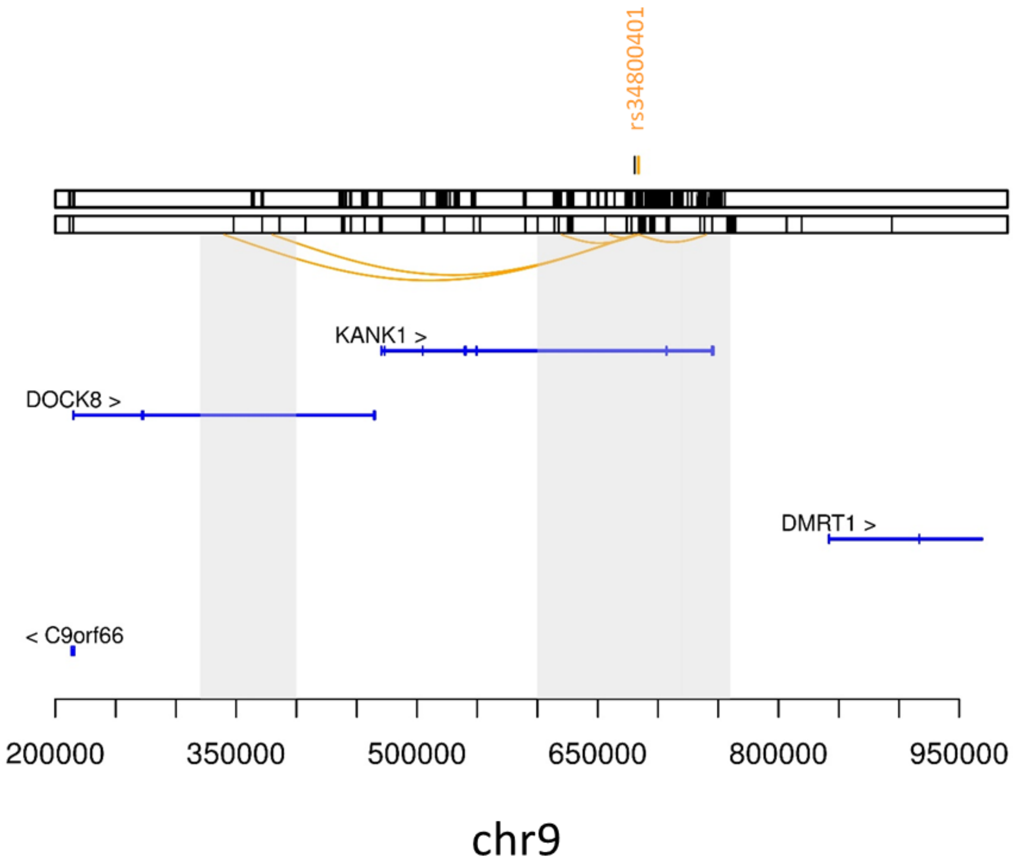

rs34800401
KANK1 >
DOCK8 >
DMRT1 >
< C9orf66
200000
350000
500000
650000
800000
950000
chr9

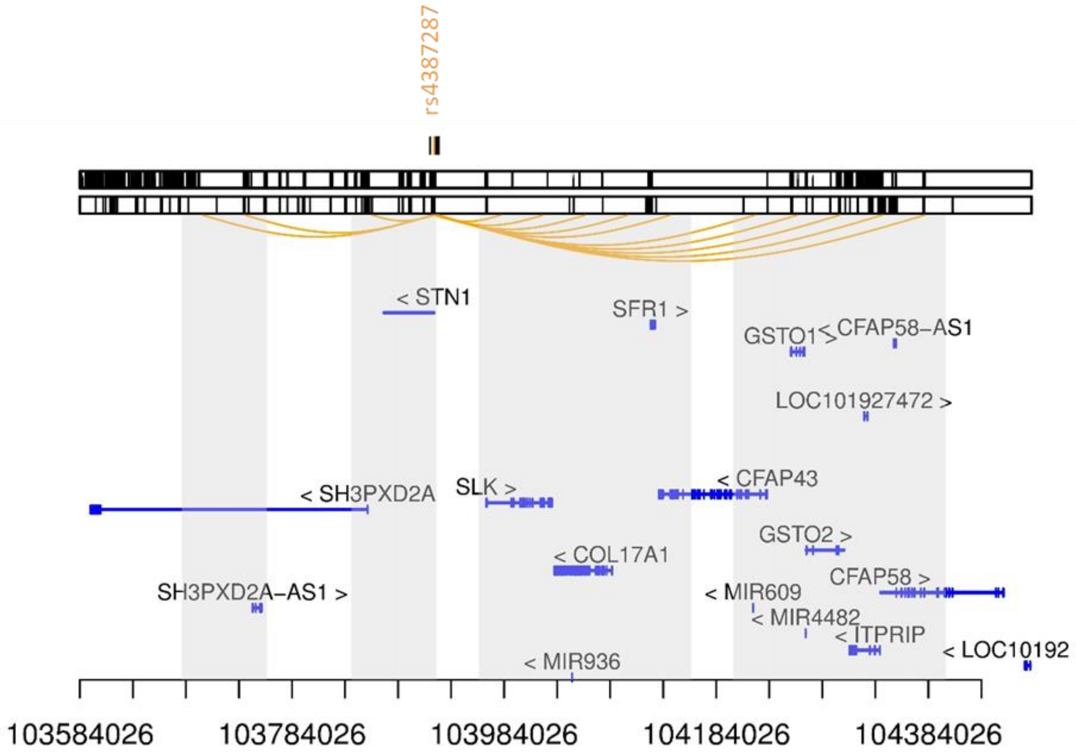

rs4387287
< STN1
SFR1 >
GSTO1 >
< CFAP58-AS1
LOC101927472 >
< SH3PXD2A
SLK >
< CFAP43
GSTO2 >
< COL17A1
CFAP58 >
SH3PXD2A-AS1 >
< MIR609
< MIR4482
< ITPRIP
< LOC10192
< MIR936
103584026
103784026
103984026
104184026
104384026

chr10

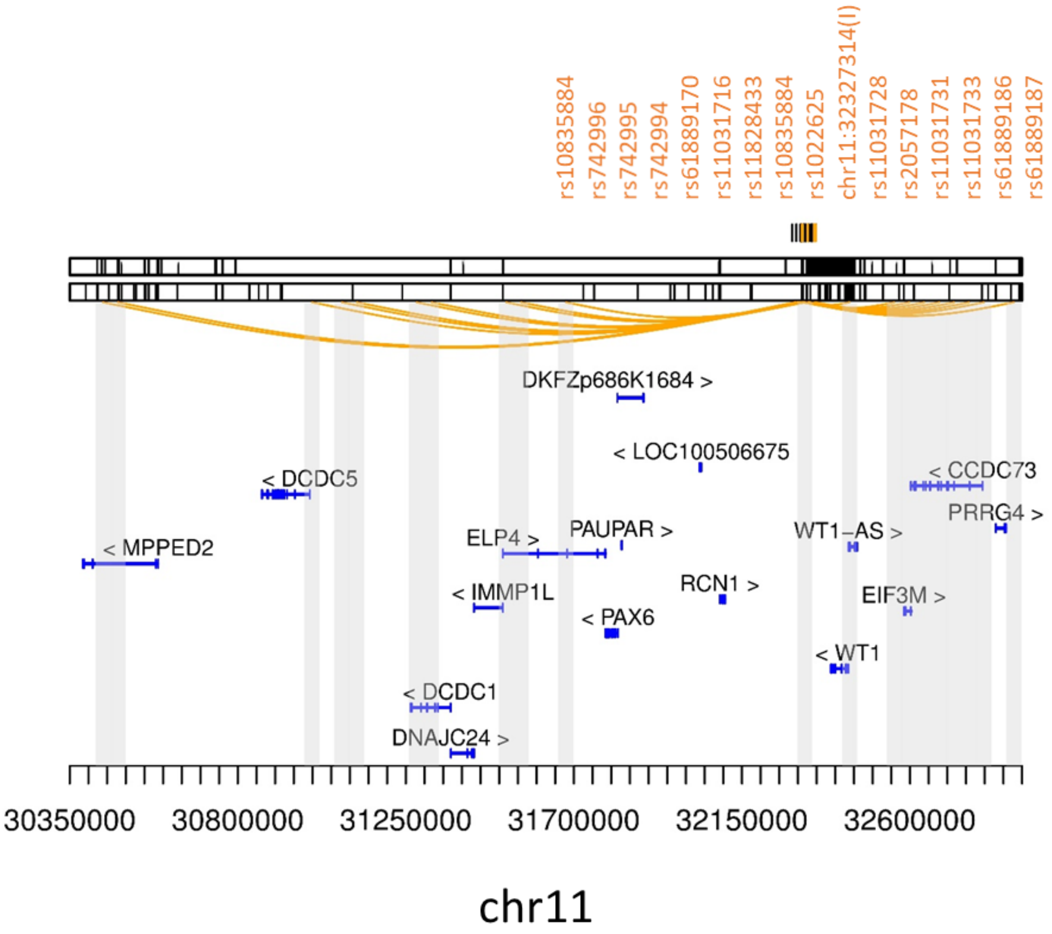
rs10835884
rs742996
rs742995
rs742994
rs61889170
rs11031716
rs11828433
rs10835884
rs1022625
chr11:32327314(I)
rs11031728
rs2057178
rs11031731
rs11031733
rs61889186
rs61889187
DKFZp686K1684 >
< LOC100506675
< CCDC73
< DCDC5
PRRG4 >
ELP4 >
PAUPAR >
WT1−AS >
< MPPED2
RCN1 >
< IMMP1L
EIF3M >
< PAX6
< WT1
< DCDC1
DNAJC24 >
30350000
30800000
31250000
31700000
32150000
32600000
chr11

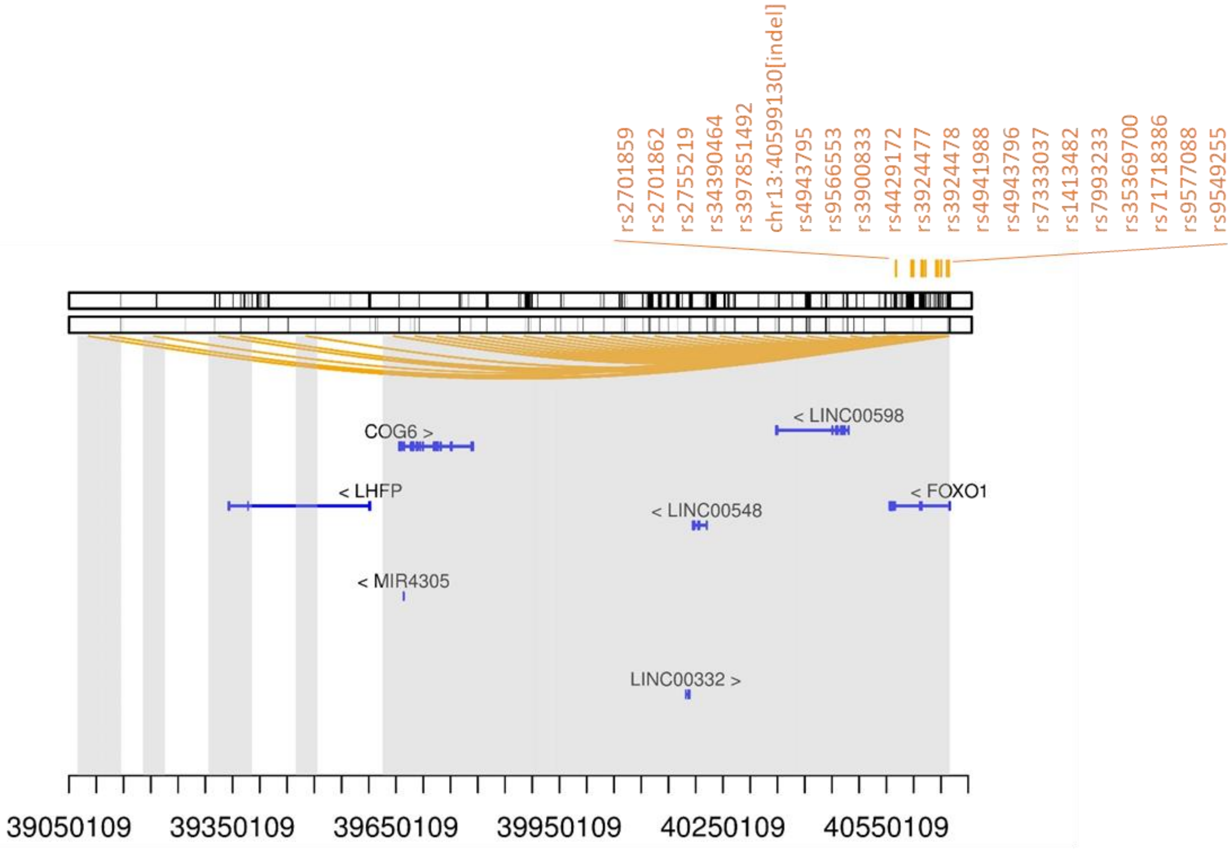

rs2701859
rs2701862
rs2755219
rs34390464
rs397851492
chr13:40599130[indel]
rs4943795
rs9566553
rs3900833
rs4429172
rs3924477
rs3924478
rs4941988
rs4943796
rs7333037
rs1413482
rs7993233
rs35369700
rs71718386
rs9577088
rs9549255
COG6 >
< LINC00598
< LHFP
< FOXO1
< LINC00548
< MIR4305
LINC00332 >
39050109
39350109
39650109
39950109
40250109
40550109

chr13

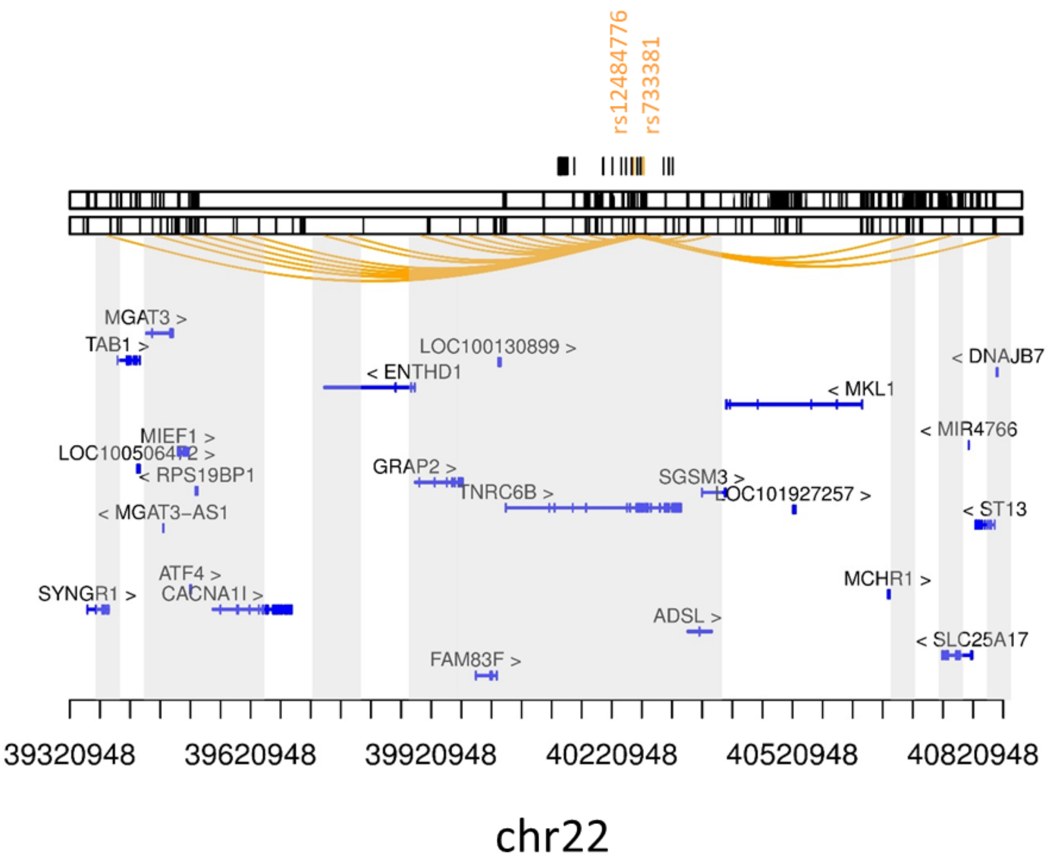

Supplementary figure 2. Leiomyoma risk variants intersecting with regulatory regions and their candidate target genes.
