# Supplementary material for: Variants associating with uterine leiomyoma highlight genetic background shared by various cancers and hormone-related traits
Source: Nat Commun. 2018 Sep 7;9:3636. doi: 10.1038/s41467-018-05428-6 (PMC6128903; doi:10.1038/s41467-018-05428-6)
Supplement: Supplementary file 3 — Description of Additional Supplementary Files [file 41467_2018_5428_MOESM3_ESM.pdf]

Description of Additional Supplementary Files

File Name: Supplementary Data 1

Description: P value thresholds for genome-wide significance for variants in different annotations classes

File Name: Supplementary Data 2

Description: Association results for variants reaching genome-wide significance in meta-analysis of leiomyoma

File Name: Supplementary Data 3

Description: Association results for 21 lead variants in meta-analysis of Icelandic and UK GWAS' of leiomyoma with summary of variants reported for other phenotypes at the loci.

File Name: Supplementary Data 4

Description: Association results for variants with P value $< 5 \times 10^{-8}$ in meta-analysis of leiomyoma

File Name: Supplementary Data 5

Description: Phenotypes tested for association with lead SNPs

File Name: Supplementary Data 6

Description: Association of leiomyoma risk variants with cancer and hormone-related traits in Iceland

File Name: Supplementary Data 7

Description: Association of leiomyoma with reported endometriosis variants

File Name: Supplementary Data 8

Description: Association results for variants correlated with rs7913069 (OBFC1 locus) in the Japanese population

File Name: Supplementary Data 9

Description: Association results for leiomyoma variants in a meta-analysis of endometrial cancer in the Icelandic and UKB populations.

File Name: Supplementary Data 10

Description: Association between reported endometrial cancer variants and meta-analysis of leiomyoma

File Name: Supplementary Data 11

Description: Summary of functional annotation of the 14 non-coding leiomyoma risk loci
