# Supplementary material for: Variants associating with uterine leiomyoma highlight genetic background shared by various cancers and hormone-related traits
Source: Nat Commun. 2018 Sep 7;9:3636. doi: 10.1038/s41467-018-05428-6 (PMC6128903; doi:10.1038/s41467-018-05428-6)
Supplement: Supplementary file 6 — Supplementary Data 3 [file 41467_2018_5428_MOESM6_ESM.docx]

| **Marker Name**  **Pos hg38^1^** | **coding effect** | **EAF** | **EA/OA** | **Gene** | **Pop.** | **OR^2^ (95%CI)** | **P** | **Info** | **Phet** | **Correlated variants reported for other phenotypes^3^** |
| --- | --- | --- | --- | --- | --- | --- | --- | --- | --- | --- |
| rs10917151  chr1:22096228 | downstr. | 0.201 | A/G | *CDC42/*  WNT4 | Ice | 1.16 (1.11, 1.22) | 3.0E-09 | 1.00 |  | r2=1 with rs12037376 (EM)(*32*), r2=0.81 with rs7521902 (BMD)(*33*), r2=0.97 with rs3820282 (OC)(*35*) |
|  |  |  |  |  | UKB | 1.10 (1.06, 1.14) | 8.5E-07 | 1.00 |  |  |
|  |  |  |  |  | **Meta** | **1.12 (1.09, 1.16)** | **5.1E-14** |  | **0.10** |  |
| rs148143917  chr2:11524625 | upstream | 0.019 | C/A | *GREB1* | Ice | 0.70 (0.60, 0.81) | 4.5E-06 | 0.99 |  | r2=0.006 with rs11674184 (EM)(*32*), r2=0.023 with rs77294520 (EM)(*32*)  r2=0.003withrs113084984(BrCa)(*37*) |
|  |  |  |  |  | UKB | 0.77 (0.68, 0.87) | 2.6E-05 | 0.86 |  |  |
|  |  |  |  |  | **Meta** | **0.74 (0.67, 0.82)** | **8.1E-10** |  | 0.34 |  |
| rs10929757  chr2:11562535 | missense Asn77Thr | 0.410 | A/C | *GREB1* | Ice | 0.92 (0.88, 0.96) | 1.1E-04 | 1.00 |  | r2=0.17 with rs11674184 (EM)(*32*), r2=0.02 with rs77294520 (EM)(*32*)  r2=0.44 with rs113084984(BrCa)(*37*) |
|  |  |  |  |  | UKB | 0.92 (0.89, 0.95) | 1.8E-08 | 0.98 |  |  |
|  |  |  |  |  | **Meta** | **0.92 (0.90, 0.94)** | **8.1E-12** |  | **1.00** |  |
| rs479404  chr3:27321573 | intron | 0.322 | C/T | *NEK10* | Ice | 1.11 (1.07, 1.16) | 1.6E-06 | 1.00 |  | r2=0.27 with rs653465 (BrC) (*37*), r2=0.13 with rs4973768 (BrC)(*37*) |
|  |  |  |  |  | UKB | 1.08 (1.04, 1.11) | 6.6E-06 | 0.99 |  |  |
|  |  |  |  |  | **Meta** | **1.09 (1.06, 1.12)** | **8.9E-11** |  | **0.26** |  |
| rs765333492  chr4:53021103 | intron | 0.003 | C/T | *SCFD2* | Ice | 2.76 (2.11, 3.6) | 2.3E-13 | 0.98 |  | No reports |
|  |  |  |  |  | UKB | NA | NA | NA |  |  |
|  |  |  |  |  | **Meta** | **2.76 (2.11, 3.6)** | **2.3E-13** |  | **1.00** |  |
| rs2202282  chr4:69768723 | intergenic | 0.485 | T/C | *SULT1E1* | Ice | 1.11 (1.06-1.15) | 8.0E-07 | 1.00 |  | No reports |
|  |  |  |  |  | UKB | 1.08 (1.05-1.11) | 1.1E-07 | 1.00 |  |  |
|  |  |  |  |  | **Meta** | **1.09 (1.07-1.12)** | **6.5E-13** |  | **0.35** |  |
| rs10069690  chr5:1279675 | intron | 0.259 | T/C | *TERT* | Ice | 1.11 (1.06, 1.16) | 1.3E-05 | 1.00 |  | Variant also reported for ThC(*14*), BrC(*15,16*), CLL (*17*), TeC, PrC, UBC, PaC and glioma (all in ref *18*) |
|  |  |  |  |  | UKB | 1.13 (1.1, 1.17) | 4.0E-14 | 1.00 |  |  |
|  |  |  |  |  | **Meta** | **1.12 (1.1, 1.15)** | **3.6E-18** |  | **0.43** |  |
| rs58415480  chr6:15224113 | intron | 0.142 | G/C | *SYNE1/*  *ESR1* | Ice | 1.19 (1.13, 1.26) | 3.7E-10 | 1.00 |  | r2=0.94 with rs71575922 (EM)(*32*) |
|  |  |  |  |  | UKB | 1.17 (1.13, 1.21) | 3.2E-15 | 0.99 |  |  |
|  |  |  |  |  | **Meta** | **1.18 (1.14, 1.22)** | **9.0E-24** |  | **0.54** |  |
| rs73639400  chr9:683423 | intron | 0.142 | C/T | *KANK1* | Ice | 1.10 (1.03-1.16) | 2.2E-03 | 1.00 |  | No reports of variants with r2 > 0.01 |
|  |  |  |  |  | UKB | 1.14 (1.10-1.19) | 7.5E-10 | 0.99 |  |  |
|  |  |  |  |  | **Meta** | **1.12 (1.09-1.16)** | **7.9E-12** |  | **0.23** |  |
| rs7030354  chr9:804231 | intergenic | 0.404 | T/C | *DMRT1* | Ice | 1.07 (1.03, 1.16) | 1.1E-03 | 1.00 |  | No reports of variants with r2 > 0.1 |
|  |  |  |  |  | UKB | 1.13 (1.10, 1.16) | 5.8E-16 | 1.00 |  |  |
|  |  |  |  |  | **Meta** | **1.11 (1.08, 1.14)** | **2.0E-17** |  | **0.03** |  |
| rs7907606  chr10:103920874 | upstream | 0.155 | G/T | *OBFC1* | Ice | 1.1 (1.04, 1.15) | 1.0E-03 | 1.00 |  | r2=0.001 with rs7913069 (LM in Japanese)(*7*). Variant reported for BCC(*25*). Correl. variants reported for multiple cancers and TL (*26-31*) |
|  |  |  |  |  | UKB | 1.1 (1.06, 1.15) | 8.0E-07 | 1.00 |  |  |
|  |  |  |  |  | **Meta** | **1.1 (1.07, 1.14)** | **3.1E-09** |  | **0.86** |  |
| rs11246001  chr11:210899 | upstream | 0.044 | T/C | *BET1L* | Ice | 0.77 (0.69, 0.86) | 1.7E-06 | 1.00 |  | r2=1.00 with rs2280543 (LM in Japanese)(*7*) |
|  |  |  |  |  | UKB | 0.84 (0.79-0.90) | 3.0E-07 | 1.00 |  |  |
|  |  |  |  |  | **Meta** | **0.82 (0.78-0.87)** | **5.2E-12** |  | **0.18** |  |
| rs507139  chr11:225196 | intron | 0.082 | A/G | *SIRT3* | Ice | 0.84 (0.78, 0.90) | 2.0E-06 | 1.00 |  | r2=0.005 with rs2280543 (LM) in Japanese)(*7*) |
|  |  |  |  |  | UKB | 0.83 (0.79, 0.88) | 1.3E-11 | 0.98 |  |  |
|  |  |  |  |  | **Meta** | **0.83 (0.80, 0.87)** | **1.4E-16** |  | **0.79** |  |
| rs11031731  chr11:32343884 | intergenic | 0.158 | A/G | *WT1* | Ice | 1.14 (1.08-1.20) | 2.3E-06 | 1.00 |  | No reports |
|  |  |  |  |  | UKB | 1.14 (1.10-1.18) | 4.7E-11 | 1.00 |  |  |
|  |  |  |  |  | **Meta** | **1.14 (1.10-1.17)** | **5.4E-16** |  | **0.96** |  |
| rs1800057  chr11:108272729 | missense Pro1054Arg | 0.020 | G/C | *ATM* | Ice | 1.27 (1.11, 1.46) | 7.0E-04 | 1.00 |  | Variant also reported for CLL(*22*), PrC(*23*), RCC(*24*) |
|  |  |  |  |  | UKB | 1.29 (1.18, 1.41) | 1.1E-08 | 1.00 |  |  |
|  |  |  |  |  | **Meta** | **1.28 (1.19, 1.38)** | **3.1E-11** |  | **0.85** |  |
| rs1801516^3^  chr11:108304735 | missense Asp1853Asn | 0.152 | A/G | *ATM* | Ice | 0.93 (0.88, 0.98) | 1.1E-02 | 1.00 |  | Variant also reported for CMM(*19*) and response to chemo- and radiotherapy(*20,21*) |
|  |  |  |  |  | UKB | 0.91 (0.88, 0.95) | 1.7E-06 | 1.00 |  |  |
|  |  |  |  |  | **Meta** | **0.91 (0.88, 0.94)** | **7.4E-08** |  | **0.53** |  |
| rs117245733  chr13:40149807 | intergenic | 0.020 | A/G | *LINC0* | Ice | 1.38 (1.21, 1.57) | 1.1E-06 | 1.00 |  | No reports |
|  |  |  |  |  | UKB | 1.23 (1.16, 1.36) | 3.3E-05 | 0.92 |  |  |
|  |  |  |  |  | **Meta** | **1.32 (1.21, 1.44)** | **2.2E-10** |  | **0.16** |  |
| rs7986407^3^  chr13:40605661 | intron | 0.305 | G/A | *FOXO1* | Ice | 1.06 (1.02, 1.11) | 6.2E-03 | 1.00 |  | No reports |
|  |  |  |  |  | UKB | 1.08 (1.05, 1.12) | 3.1E-06 | 1.00 |  |  |
|  |  |  |  |  | **Meta** | **1.07 1.04, 1.10()** | **7.0E-08** |  | **0.49** |  |
| rs78378222  chr17:7668434 | 3’UTR | 0.018 | G/T | *TP53* | Ice | 1.83 (1.61, 2.08) | 3.4E-20 | 1.00 |  | Variant also reported for BCC, PrC, glioma, CR adenoma (all in ref *11*), glioma(*12*) and neuroblastoma*(13*) |
|  |  |  |  |  | UKB | 1.67 (1.5, 1.87) | 8.7E-19 | 0.96 |  |  |
|  |  |  |  |  | **Meta** | **1.74 (1.6, 1.89)** | **4.0E-37** |  | **0.31** |  |
| rs16991615  chr20:5967581 | missense Glu341Lys | 0.081 | A/G | *MCM8* | Ice | 1.17 (1.09, 1.25) | 3.1E-05 | 1.00 |  | Variant also reported for age at menopause(*36*) and BrCa(*37*) |
|  |  |  |  |  | UKB | 1.15 (1.09, 1.21) | 1.3E-06 | 1.00 |  |  |
|  |  |  |  |  | **Meta** | **1.16 (1.11, 1.21)** | **3.6E-10** |  | **0.99** |  |
| rs12484951  chr22:40307071 | intron | 0.252 | G/T | *TNRC6B* | Ice | 1.16 (1.11, 1.22) | 1.1E-10 | 1.00 |  | r2=0.97 with rs12484776 (LM) in Japanese(*7*) |
|  |  |  |  |  | UKB | 1.08 (1.04, 1.11) | 2.2E-05 | 1.00 |  |  |
|  |  |  |  |  | **Meta** | **1.11 (1.08, 1.14)** | **3.2E-13** |  | **0.01** |  |

**Supplementary Data 3. Association results for 21 significant lead variants in meta-analysis of leiomyoma and summary of correlated variants reported for other phenotypes at the locus.**

^1^Marker positions are according to GRCh38/hg38, ^2^Odds-ratios correspond to effect alleles, ^3^Previously reported associations of genome-wide significance with references to the respective studies in brackets, ^4^Secondary signals reaching conditional P value < 10-6 (approximately 30.000 variants tested).

Abbreviations: P Meta, P-value for fixed effects meta-analysis; OR, Odds ratio; Info, Imputation information; Phet, P-value for heterogeneity in the effect estimate between the Icelandic and UK Biobank data; EAF, effect allele frequency; EA, effect allele; OA, other allele; EM, endometriosis; BMD. bone mineral density; OC, ovarian cancer; BrC, breast cancer; TeC, testicular cancer; LM, leiomyoma; PrC, prostate cancer; UBC, urinary bladder cancer; ThC, thyroid cancer; PaC, pancreatic cancer; BCC, basal cell carcinoma; CLL, chronic lymphocytic leukemia: RCC, renal cell carcinoma; CMM, cutaneous malignant melanoma; CR adenoma, colorectal adenoma; TL, telomere length.
