# Supplementary material for: Variants associating with uterine leiomyoma highlight genetic background shared by various cancers and hormone-related traits
Source: Nat Commun. 2018 Sep 7;9:3636. doi: 10.1038/s41467-018-05428-6 (PMC6128903; doi:10.1038/s41467-018-05428-6)
Supplement: Supplementary file 14 — Supplementary Data 11 [file 41467_2018_5428_MOESM14_ESM.docx]

| **Chrom** | **Pos** | **Lead variant** | **H3K27-acetylation region^1^** | **DHS region^1^** | **Chromatin interactions (Hi-C) between regulatory variants and genes^2^** | **Gene promoter^3^** | **No in LD class** |
| --- | --- | --- | --- | --- | --- | --- | --- |
| chr1 | 22096228 | rs10917151 | rs3768579, rs3754496, rs7412010 |  | **CDC42**, ZBTB40, USP48, RAP1GAP, WNT4, MIR4418, EPHA8, MIR6127, C1QA, C1QB, C1QC, EPHB2, MIR4684, KDM1A, MIR3115, MIR4419A, LUZP1, HTR1D | CDC42 | 37 |
| chr2 | 11524625 | rs148143917 | rs148143917 |  | PDIA6, GREB1, MIR4429 |  | 5 |
| chr3 | 27321573 | rs479404 |  |  |  |  | 51 |
| chr4 | 53021103 | rs765333492 |  |  |  |  | 2 |
| chr4 | 69768723 | rs2202282 |  |  |  | SULT1B1 | 8 |
| chr5 | 1279675 | rs10069690 |  |  |  |  | 1 |
| chr6 | 152241136 | rs58415480 | rs58415480, rs71575922 | rs58415480, rs71575922 | ***ESR1***, **SYNE1**, MYCT1, ARMT1, CCDC170, RMND1, VIP |  | 2 |
| chr9 | 683423 | rs73639400 | rs73639400, rs12004436, rs10975820 |  | **DOCK8**, KANK1 |  | 4 |
| chr9 | 804231 | rs7030354 |  | rs34800401 | **DOCK8**, KANK1, DMRT1 |  | 21 |
| chr10 | 103920874 | rs7907606 | rs4387287 |  | SH3PXD2A, STN1, SLK, COL17A1, MIR936, SFR1, CFAP43, MIR609, GSTO1, MIR4482, GSTO2, ITPRIP, LOC101927472, CFAP58 | STN1 | 7 |
| chr11 | 225196 | rs507139 |  | rs5789177, rs3839961 |  | SCGB1C1, ODF3 | 34 |
| chr11 | 210899 | rs11246001 |  |  |  |  | 36 |
| chr11 | 32343884 | rs11031731 | rs10835884, rs742996, rs742995, rs742994, rs61889170, rs11031716, rs11828433, chr11:32327314[I], rs11031728, rs2057178, rs11031731, rs11031733, rs61889186, rs61889187 | rs10835884, rs1022625, rs11031716, rs11828433 | *MPPED2*, **WT1**, QSER1, DCDC1, DCDC5, ELP4, IMMP1L, CCDC73, EIF3M |  | 44 |
| chr13 | 40149807 | rs117245733 | rs117245733 | rs117245733 | COG6, LINC00332, LINC00548, LINC00598, **FOXO1**, **MRPS31**, SLC25A15, TPTE2P5, MIR621, SUGT1P3, ELF1 |  | 1 |
| chr13 | 40605661 | rs7986407 | rs2701859, rs2701862, rs2755219, rs34390464,rs397851492, chr13:40599130[l], rs4943795, rs9566553, rs3900833, rs4429172, rs3924477, rs3924478, rs4941988, rs4943796, rs7333037, rs1413482, rs7993233, rs35369700,rs71718386, rs9577088, rs9549255, chr13:40664806[I], chr13:40664807[I], rs6563841 | rs3900833, chr13:40664806[l] chr13:40664807[I] | **FOXO1**, **LHFP**, COG6, MIR4305, LINC00332, LINC00548, LINC00598 |  | 134 |
| chr22 | 40307071 | rs12484951 | rs12484776, rs733381 | rs12484776 | ADSL, ATF4, CACNA1I, DNAJB7, ENTHD1, FAM83F, GRAP2, MGAT3, MIEF1, RPS19BP1, SGSM3, SLC25A17, ST13, SYNGR1, TAB1, TNRC6B, XPNPEP3, LOC100130899 |  | 48 |

**Table 2**. Summary of functional annotation of the 14 non-coding leiomyoma risk loci

^1^ Variants in the same LD class as the lead variants (R-squared > 0.80) annotated for regions characterized as H3K27-acetylated or DHS (Dnase hypersensitivity sites). Variants without rs names are denoted by location in hg38, [I] indicates that the variant is an insertion or deletion

^2^Chromatin interactions are reported for refseq genes found in contact with regulatory regions in uterine tissue wherein variants within an LD class reside, i.e. H3K27-acetylated or DHS regions. Genes found recurrently mutated in cancer (NCG5.0) marked in bold. Genes identifed as tissue-specific for uterus, based on Human Protein Atlas database (v18) are marked in italics, i.e. *ESR1* and *MPPED2*.

^3^Gene promoters intersecting with variants in LD class. Promoters defined here as < 1kb from gene transcription start sites (refseq annotation)
